# Identification and characterization of VapBC toxin–antitoxin system in *Bosea* sp. PAMC 26642 isolated from Arctic lichens

**DOI:** 10.1261/rna.078786.121

**Published:** 2021-11

**Authors:** Hyerin Jeon, Eunsil Choi, Jihwan Hwang

**Affiliations:** 1 Department of Microbiology, Pusan National University, Busan 46241, Republic of Korea; 2 Microbiological Resource Research Institute, Pusan National University, Busan 46241, Republic of Korea

**Keywords:** toxin–antitoxin system, VapBC, endoribonuclease, *Bosea*, extremophile

## Abstract

Toxin–antitoxin (TA) systems are genetic modules composed of a toxin interfering with cellular processes and its cognate antitoxin, which counteracts the activity of the toxin. TA modules are widespread in bacterial and archaeal genomes. It has been suggested that TA modules participate in the adaptation of prokaryotes to unfavorable conditions. The *Bosea* sp. PAMC 26642 used in this study was isolated from the Arctic lichen *Stereocaulon* sp. There are 12 putative type II TA loci in the genome of *Bosea* sp. PAMC 26642. Of these, nine functional TA systems have been shown to be toxic in *Escherichia coli*. The toxin inhibits growth, but this inhibition is reversed when the cognate antitoxin genes are coexpressed, indicating that these putative TA loci were bona fide TA modules. Only the BoVapC1 (AXW83_01405) toxin, a homolog of VapC, showed growth inhibition specific to low temperatures, which was recovered by the coexpression of BoVapB1 (AXW83_01400). Microscopic observation and growth monitoring revealed that the BoVapC1 toxin had bacteriostatic effects on the growth of *E. coli* and induced morphological changes. Quantitative real time polymerase chain reaction and northern blotting analyses showed that the BoVapC1 toxin had a ribonuclease activity on the initiator tRNA^fMet^, implying that degradation of tRNA^fMet^ might trigger growth arrest in *E. coli*. Furthermore, the BoVapBC1 system was found to contribute to survival against prolonged exposure at 4°C. This is the first study to identify the function of TA systems in cold adaptation.

## INTRODUCTION

Toxin–antitoxin systems (TA systems) are genetic modules composed of a toxin, which is engaged in the regulation of vital processes, and an antitoxin that blocks the activities of the toxin (Mruk and Kobayashi 2014; Kedzierska and Hayes 2016). TA systems are widespread in prokaryotes. The recent availability of large amounts of genome sequencing data and the development of sophisticated bioinformatics tools have revealed that this wide distribution is the result of horizontal gene transfer (Van Melderen and De Bast 2009; Fozo et al. 2010; Habib et al. 2018; Akarsu et al. 2019). A toxin and its antidote antitoxin genes are typically organized in a bi-cistronic operon and are transcriptionally coexpressed (Ogura and Hiraga 1983; Mruk and Kobayashi 2014). Under normal growth conditions, the coexpressed antitoxin inhibits the expression or activity of the toxin. However, under stress conditions, labile antitoxins are degraded by RNases or proteases (Lon or Clp), releasing and activating the toxin (Roberts et al. 1994; Van Melderen et al. 1994; Lehnherr and Yarmolinsky 1995; Mikkelsen and Gerdes 1997; Brantl and Jahn 2015; Muller et al. 2016; Muthuramalingam et al. 2016).

Toxins are always proteins, whereas an antitoxin can be either a protein or a regulatory RNA (Short et al. 2018; Horesh et al. 2020). TA systems are classified into six types (I–VI) according to the biochemical nature of the antitoxin and the mechanism of regulation of the toxin by the antitoxin (Lobato-Marquez et al. 2016; Kang et al. 2018; Kato et al. 2019; Horesh et al. 2020). In types I and III, the antitoxins are noncoding RNAs that directly bind to toxin mRNAs (type I) or proteins (type III) (Gerdes et al. 1986; Fineran et al. 2009; Brantl and Jahn 2015). The antitoxins of types II and IV TA systems are proteins which directly interact with toxin proteins to inhibit the activity of toxins (type II), or indirectly obstruct toxins by binding to the target of the toxin (type IV) (Tam and Kline 1989; Masuda et al. 2012). Type V antitoxin has ribonuclease activity, and cleaves the mRNA of the toxin (Wang et al. 2012), while type VI antitoxin induces the degradation of a toxin by attracting the appropriate protease (Aakre et al. 2013).

Among the six types of TA systems, the type II TA system is ubiquitous in prokaryotic genomes (Leplae et al. 2011). Based on amino acid sequence similarity, toxins of the type II TA system can be further classified into nine superfamilies: RelE/ParE, MazF/CcdB, HicA, VapC, HipA, FicT/Doc, AtaT/TacT, Zeta, and MbcT (Zhang et al. 2020). Many of the type II toxins are ribonucleases that cleave various targets of mRNAs, rRNAs, or tRNAs, thereby resulting in inhibition of translation (Kang et al. 2018). In a type II TA system, its expression is auto-regulated by the binding of the antitoxin to the promoter, repressing transcription (Black et al. 1994; de la Hoz et al. 2000; Afif et al. 2001; Santos-Sierra et al. 2002; Zhang et al. 2003; Smith and Magnuson 2004; Gerdes et al. 2005; Cataudella et al. 2012). The affinity of an antitoxin for its own promoter can be strengthened by forming a complex with the toxin (Magnuson and Yarmolinsky 1998; Afif et al. 2001; Li et al. 2008; Overgaard et al. 2008; Garcia-Pino et al. 2010). This regulation, therefore, is typically controlled by the stoichiometric ratio of toxins and antitoxins, and is called conditional cooperativity (Afif et al. 2001; Overgaard et al. 2008; Garcia-Pino et al. 2010; Boggild et al. 2012; Cataudella et al. 2012; Winther and Gerdes 2012).

The VapBC (virulence associated proteins) TA systems, consisting of toxin VapC and antitoxin VapB, are the largest family of type II TA modules (Pullinger and Lax 1992; Pandey and Gerdes 2005; Sevin and Barloy-Hubler 2007). Multiple copies of *vapBC* loci are found in many prokaryotic genomes (Pandey and Gerdes 2005). For instance, the genomes of *Mycobacterium tuberculosis* and a thermophilic archaeon, *Sulfolobus solfataricus* encode 50 and 25 *vapBC* TA modules, respectively (Cooper et al. 2009; Ramage et al. 2009; Gupta et al. 2017). The VapC toxins belong to the PIN (PilT amino terminus) domain protein family, which shares structural similarity with the bacteriophage T4 RNase H, and shows ribonuclease activity (Arcus et al. 2011; Xu et al. 2016). With this ribonuclease domain, VapC toxins cleave primarily stable RNA species such as tRNA and rRNA in a sequence-specific manner (Winther and Gerdes 2011; Winther et al. 2013).

*Bosea* is a gram-negative bacterium belonging to the class α-proteobacteria, family *Bradyrhizobiaceae*, which has been isolated from a range of environments such as soils, sediments, and hospital water (Das et al. 1996; La Scola et al. 2003). A unique feature of *Bosea* is its ability to oxidize thiosulfate (Das et al. 1996). *Bosea* sp. PAMC 26642, used in this study, was isolated from the Arctic lichen *Streocaulon* sp. (Kang et al. 2016). *Bosea* sp. PAMC 26642 has 12 type II TA systems, considerably more than *Bosea* species inhabiting warmer environments. Of note, among the eight *Bosea* species for which complete genome sequences are available, four do not have TA systems, *Bosea vestrisii* has two TA systems, *Bosea caraganae* has two, and *Bosea psychrotolerans* has five. Likewise, *Mycobacterium tuberculosis,* which is exposed to hypoxia, oxidative stress, limited nutrient, and low pH, during macrophage infection (Wu et al. 2012), also has an enormous number of TA loci (approximately 90), whereas nonpathogenic mycobacteria possess relatively few TA loci (Ramage et al. 2009). Among them, PezT (Tandon et al. 2019), three MazFs (Tiwari et al. 2015), three RelEs (Singh et al. 2010), and six VapCs (Rengarajan et al. 2005; Agarwal et al. 2020; Sharma et al. 2020) toxins are critical for adaptation to various stresses such as oxidative stress, nutrient deprivation, drugs, or the internal macrophage environment.

We hypothesized that the abundance of type II TA systems in *Bosea* sp. PAMC 26642 may be related to adaptation of the bacterium to the cold environment of the Arctic. In this study, we identified 12 putative type II TA modules in the *Bosea* sp. PAMC 26642 genome, including five VapBC homologs, AXW83_01400-AXW83_01405, AXW83_06915-AXW83_06920, AXW83_RS26480-AXW83_RS08500, AXW83_11460-AXW83_11465, and AXW83_12680-AXW83_12685, using a web-based tool (Xie et al. 2018). The growth inhibition caused by these TAs was examined in *Escherichia coli*, and nine of the toxins were found to have an inhibitory effect on the growth of *E. coli*, which was neutralized by their cognate antitoxins. Among those nine TA systems, the expression of the BoVapC1 (AXW83_01405) toxin inhibited growth only at low temperatures by a bacteriostatic mechanism, and this growth inhibition was recovered by the coexpression of BoVapB1 (AXW83_01400). Furthermore, we demonstrated that BoVapC1 possesses cleavage activity against tRNA^fMet^ using quantitative real time polymerase chain reaction (qRT-PCR) and northern blotting, which may cause growth inhibition and increase survivability of *E. coli* at 4°C.

## RESULTS

### Evaluation of toxic effects of 12 type II TA modules in *Bosea* sp. PAMC 26642

**FIGURE 1.**
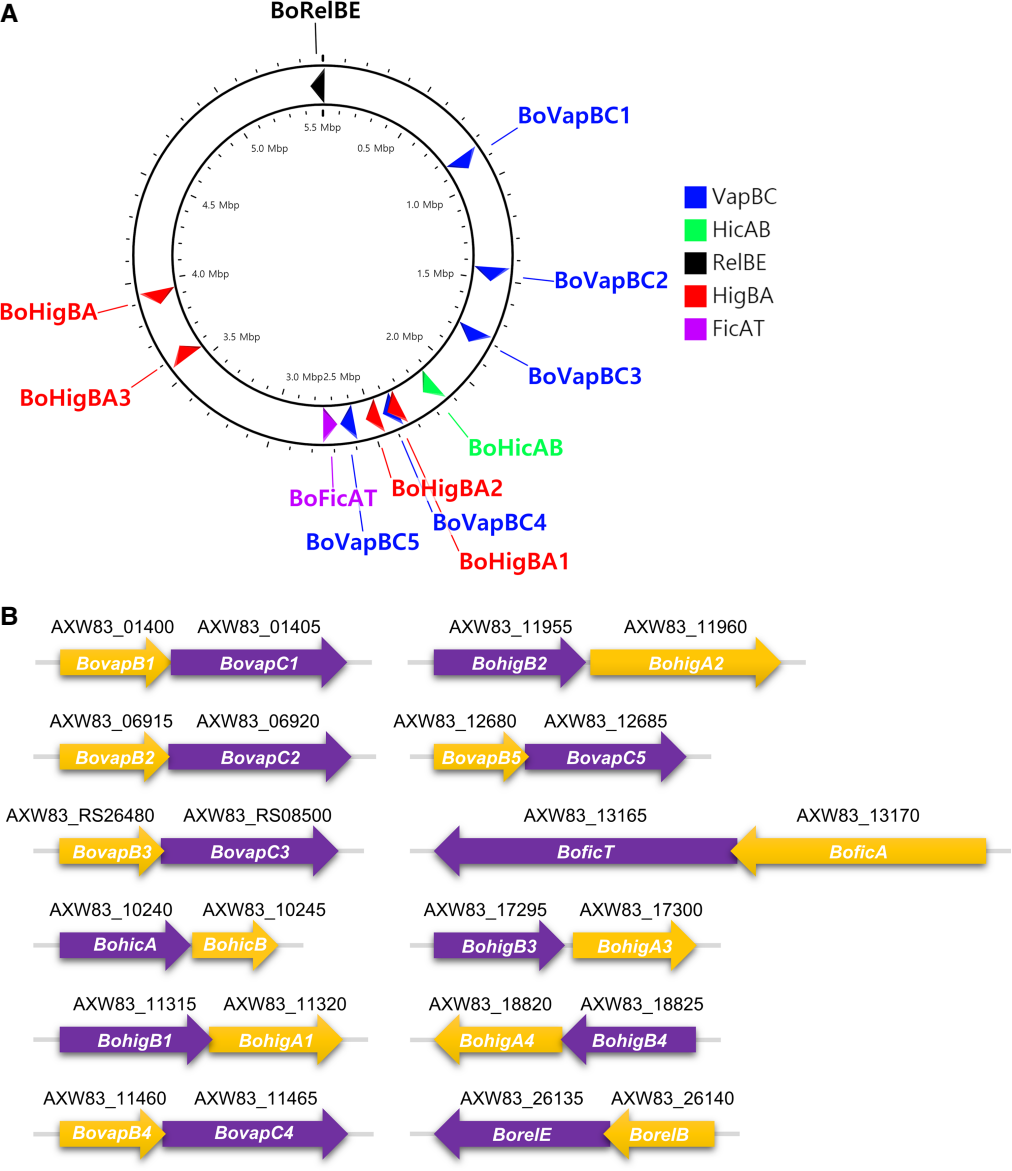
Type II TA modules of *Bosea* sp. PAMC 26642. (*A*) A schematic map of the *Bosea* sp. PAMC 26642 genome encoding 12 putative type II TA systems. A circular genome map was drawn using CG view (Stothard and Wishart 2005). (*B*) A schematic presentation of *Bosea* sp. PAMC 26642 type II TA genetic arrangements. The locus tags of the genes are shown *above* the arrows. Purple and yellow arrows indicate toxins and antitoxins, respectively.

The 12 putative type II TA systems in the *Bosea* sp. PAMC 26642 genome were identified using TAfinder (Xie et al. 2018). They include five VapBCs (BoVapBC1; AXW83_01400-AXW83_01405, BoVapBC2; AXW83_06915-AXW83_06920, BoVapBC3; AXW83_RS26480-AXW83_RS08500, BoVapBC4; AXW83_11460-AXW83_11465, and BoVapBC5; AXW83_12680-AXW83_12685), four HigBAs (BoHigBA1; AXW83_11315-AXW83_11320, BoHigBA2; AXW83_11955-AXW83_11960, BoHigBA3; AXW83_17295-AXW83_17300, and BoHigBA4; AXW83_18825-AXW83_18820), one RelBE (BoRelBE; AXW83_26140-AXW83_26135), one HicAB (BoHicAB; AXW83_10240-AXW83_10245), and one FicAT (BoFicAT; AXW83_13170-AXW83_13165) (Table 1; Fig. 1).

**TABLE 1.**
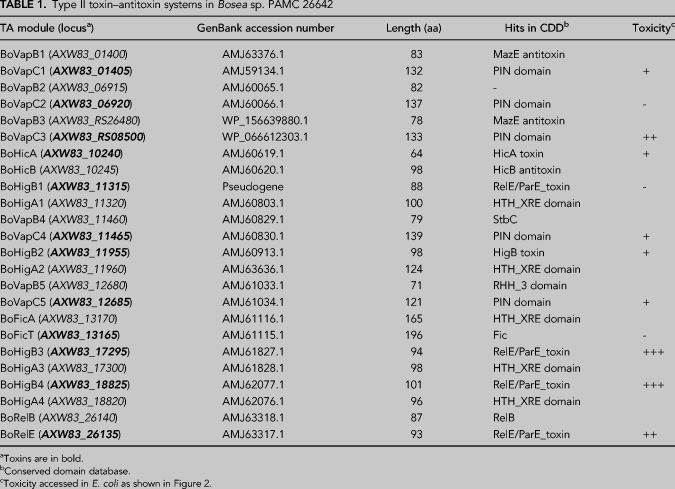
Type II toxin–antitoxin systems in *Bosea* sp. PAMC 26642

**FIGURE 2.**
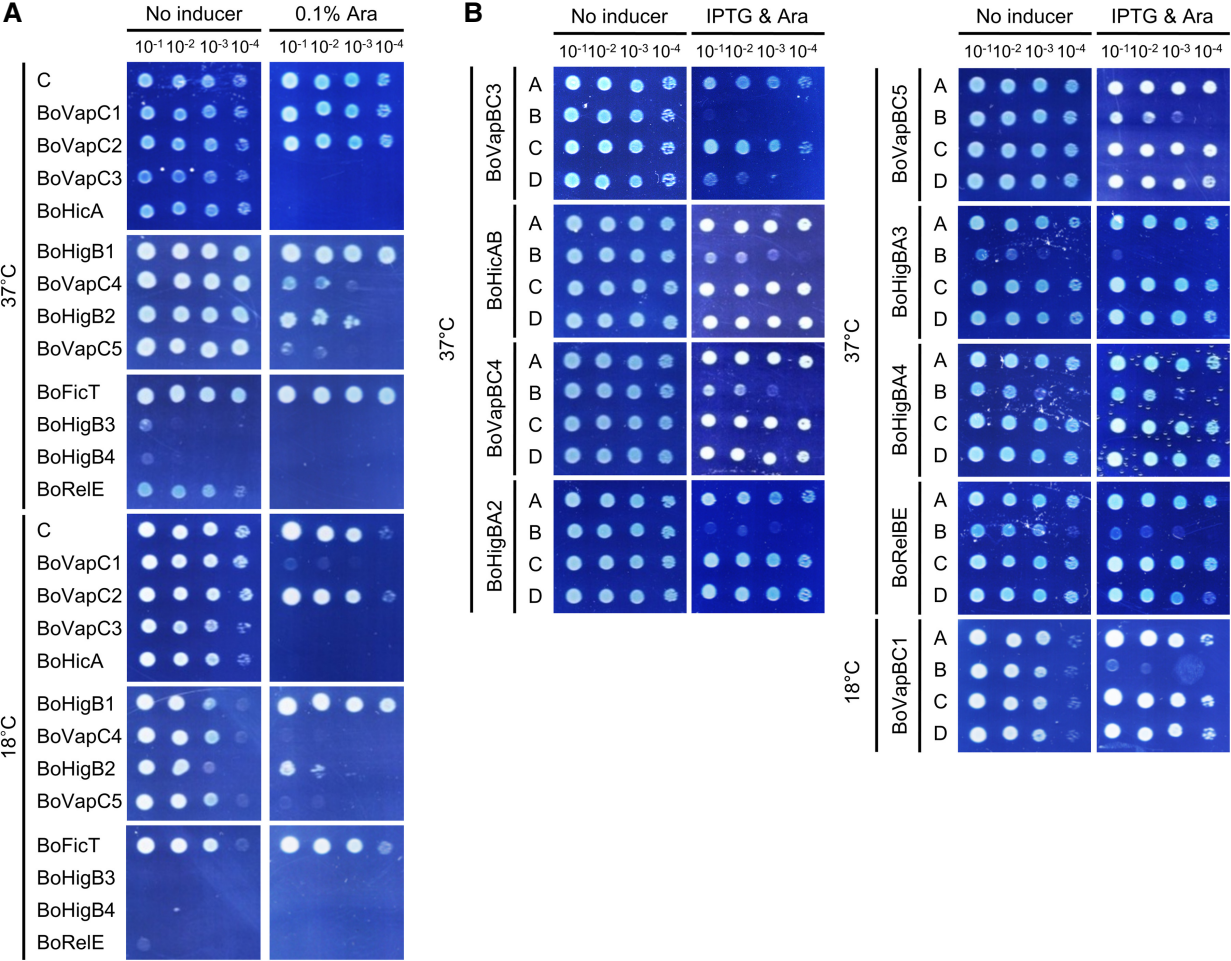
Phenotypes caused by toxins and their cognate antitoxins in *E. coli*. (*A*) The toxic effect of nine toxins of *Bosea* sp. PAMC 26642 on *E. coli* growth. *E. coli* BL21(DE3) cells transformed with pBAD33 or pBAD33-toxin were spotted on M9 agar plates supplemented with or without 0.1% arabinose, followed by incubation at 37°C or 18°C. C indicates pBAD33. (*B*) Neutralization of toxin effects by antitoxin expression. *E. coli* BL21(DE3) cells transformed with pET21c or pET21c-antitoxin were spotted on M9 agar plate supplemented with or without both arabinose and IPTG, and the plates were incubated at 37°C or 18°C. A; cells with pBAD33/pET21c, B; pBAD33-toxin/pET21c, C; pBAD33/pET21c-antitoxin, and D; pBAD33-toxin/pET21c-antitoxin.

To investigate the functionality of these putative toxins, the growth-inhibitory effects of the toxins on *E. coli* were assessed using spotting assays. Twelve toxin genes from *Bosea* sp. PAMC 26642 were cloned into pBAD33, which was transformed into the BL21(DE3) strain. All of the transformant colonies were scraped with M9 minimal media. They were diluted with the same media to an optical density at 600 nm (OD_600_) of 0.2 and serially diluted from 10^−1^ to 10^−4^. Each diluted bacterial suspension was spotted onto M9 agar plates with varying concentrations of arabinose, to determine the minimal concentration at which colony formation was inhibited by the expression of toxin. Eight of the 12 toxins produced a toxic effect on *E. coli* growth (BoVapC3, BoHicA, BoVapC4, BoHigB2, BoVapC5, BoHigB3, BoHigB4, and BoRelE) at both 37°C and 18°C (Fig. 2A; Supplemental Fig. S1). Transformants expressing BoHigB3 or BoHigB4 could not form colonies, even in the absence of arabinose. The toxic activity of BoVapC1 was temperature-dependent, being toxic only at 18°C. In contrast, the remaining three putative toxins (BoVapC2, BoHigB1, and BoFicT) did not inhibit colony formation at either temperature. Toxicity of these predicted toxins were further assessed in α-proteobacterium *Rhodobacter sphaeroides* 2.4.1. Expression of *BovapC5* and *BorelE* inhibited the growth of *R. sphaeroides* at both 30°C and 15°C, and BoVapC1 and BoVapC4 produced a toxic effect only at 15°C (Supplemental Fig. S2). These results indicate that BoVapC1 possesses the temperature-sensitive activity in both strains.

### Neutralization assay of nine TA modules

To examine whether the cognate antitoxins could neutralize the activities of the toxins, nine cognate antitoxin genes were cloned into the pET21c vector. Prior to the neutralization assays, the inhibitory effects of the cognate antitoxins on cell growth were investigated. pET21c-antitoxin plasmids were transformed into BL21(DE3) cells, and the transformants were spotted on M9 agar plates containing various concentrations of isopropyl β-d-1-thiogalactopyranoside (IPTG). Expression of these antitoxins did not influence the growth of *E. coli*, except for BoHigA3 and BoRelB, which have marginal negative effects on colony formation at the highest concentration of IPTG (0.1 mM) (Supplemental Fig. S3).

Next, to assess the ability of antitoxins to antagonize their cognate toxins, BL21(DE3) cells harboring pET21c or pET21c-antitoxin were transformed with pBAD33 or pBAD33-toxin and spotted on M9 agar plates with or without inducers. For the expression of toxins, different concentrations of arabinose were utilized as follows: BoVapC1; 0.1%, BoHicA, BoVapC4, BoHigB2, and BoVapC5; 0.05%, BoVapC3, BoHigB3, and BoRelE; 0.01%, and BoHigB4; 0.005%. The expression of the antitoxins was induced with 0.02 mM IPTG, except for BoHigA2, for which expression was induced with 0.1 mM IPTG.

Consistent with previous results (Fig. 2A), the colony formation of double-transformed cells was affected by the induction of toxin in the presence of arabinose (Supplemental Fig. S4). In the presence of both arabinose and IPTG, BL21(DE3) cells with pET21c and pBAD33-toxin showed defects in colony formation ability, which was reversed by coexpression of the toxin and the antitoxin in the cells harboring pET21c-antitoxin and pBAD33-toxin (Fig. 2B). These results suggest that these antitoxins inactivated the function of their respective toxins, and that these nine TA systems appeared to be bona fide TA systems.

### Characterization of the BoVapBC1 TA module

Of the nine functional toxins, only BoVapC1 exhibited low-temperature-specific growth-inhibitory activity in both *E. coli* and *R. sphaeroides*, implying that BoVapC1 may play a more regulatable role in the adaptation of *Bosea* sp. PAMC 26642 to extreme or cold environments than other toxins. Therefore, our studies were focused on the BoVapBC1 TA module.

**FIGURE 3.**
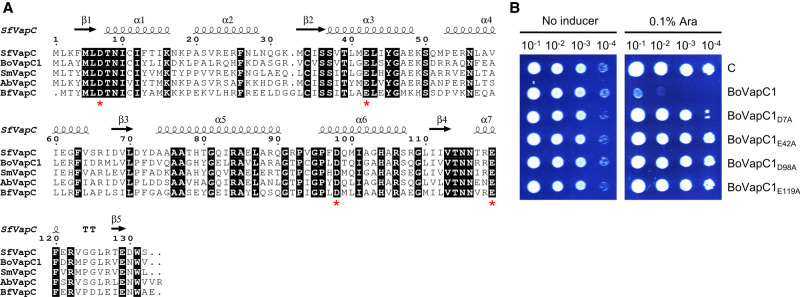
Multiple sequence alignment of BoVapC1 with previously characterized VapC homologs in other bacteria. (*A*) The primary sequence of BoVapC1 (AXW83_01405) was aligned with various VapC homologs in *S. flexneri* (Sf), *S. meliloti* (Sm), *A. bacterium* (Ab), and *B. fragilis* (Bf). The secondary structure of SfVapC is presented *above* the alignment. The acidic amino acid residues which are conserved in the active site of VapC are shown by red asterisks at the *bottom* of the alignment. The numbers correspond to the residues. The GenBank accession numbers for the VapC homologs are as follows: SfVapC; WP_000911329.1, SmVapC; WP_003527486.1, AbVapC; NDA60252.1, BfVapC; KAA4851476.1. (*B*) Effect of mutations in conserved acidic residues of BoVapC on growth-inhibitory activity. *E. coli* BL21(DE3) cells were transformed with pBAD33, pBAD33-BoVapC1, or pBAD33-BoVapC1 mutants. The transformant colonies were scraped and diluted as described in Figure 2. Bacterial suspensions were spotted on M9 agar plates supplemented with 0% and 0.1% arabinose. The plates were incubated at 18°C. C indicates pBAD33.

Using multiple sequence alignment, the amino acid sequences of BoVapB1 and BoVapC1 proteins were compared with other VapB or VapC homologs, respectively, in other bacteria such as *Shigella flexneri*, *Sinorhizobium meliloti*, *Actinobacteria bacterium*, and *Bacteroides fragilis*. As shown in Figure 3A, the toxin BoVapC1 had a high level of sequence similarity with other VapC homologs. BoVapC1 had 64%, 57%, 55%, and 50% amino acid identity with *S. meliloti*, *A. bacterium*, *S. flexneri*, and *B. fragilis*, respectively. The PIN domain in VapC harbors three to five conserved acidic amino residues, which form a negatively charged pocket for divalent metal cations and create the active site for metal-dependent ribonuclease activity (Senissar et al. 2017). These acidic residues were found to be well conserved in BoVapC1 (Fig. 3A). As shown in Figure 3B, the D7, E42, D98, or E119 residues in BoVapC1 were substituted with alanine to investigate whether the four conserved acidic residues in the active site are indispensable for its toxicity. Upon induction, the four mutants did not exhibit any growth-inhibitory activity in BL21(DE3). These results suggest that these four acidic amino acid residues play a pivotal role in the toxicity of BoVapC1, and that the growth inhibition was caused by the BoVapC1 protein.

**FIGURE 4.**
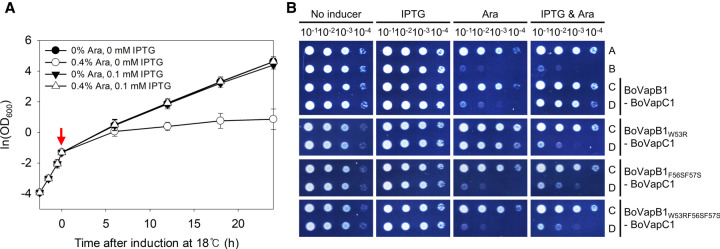
Growth inhibition by BoVapC1 and its recovery by BoVapB1. (*A*) Neutralization of BoVapC1-mediated growth inhibition by BoVapB1. BL21(DE3) cells harboring pET21c-BoVapB1 and pBAD33-BoVapC1 were cultivated as described in Materials and Methods, and OD_600_ values were measured. Red arrow indicates induction starting point. (*B*) Neutralization of BoVapC1 by BoVapB1 mutants. The BL21(DE3) cells pretransformed with pET21c, pET21c-BoVapB1, or pET21c-BoVapB1 mutants were double transformed with pBAD33 or pBAD33-BoVapC1. The neutralization assay was conducted as described in Figure 2. A; cells with pBAD33/pET21c, B; pBAD33-BoVapC1/pET21c, C; pBAD33/pET21c-BoVapB1, and D; pBAD33-BoVapC1/pET21c-BoVapB1.

To confirm that BoVapC1 inhibited cell growth in liquid media, BL21(DE3) cells harboring pBAD33 or pBAD33-BoVapC1 were grown in M9 minimal media supplemented with chloramphenicol. Arabinose (0.4%) was added for toxin expression during the early exponential phase. The growth was inhibited by BoVapC1 expression at 18°C but was not inhibited at 37°C (Supplemental Fig. S5A,B). The expression of the antitoxin BoVapB1 induced by 0.1 mM IPTG had no effect on the growth of *E. coli* (Supplemental Fig. S5C). In M9 minimal media supplemented with 0.4% arabinose and 0.1 mM IPTG, the growth defect caused by the toxin BoVapC1 was recovered by coexpression with the antitoxin BoVapB1, indicating that BoVapB1 can antagonize the activity of BoVapC1 in vivo (Fig. 4A). By primary and tertiary structure comparisons with *S. flexneri* VapBC, three aromatic amino acids (W53, F56, and F57) of BoVapB1 were predicted to interact tightly with the hydrophobic core of the VapC toxin (Supplemental Fig. S6; Dienemann et al. 2011), possibly leading to neutralization of BoVapC1. As shown in Figure 4B, the BoVapB1 mutants failed to antagonize the BoVapC1 toxicity, suggesting that neutralization by BoVapB1 expression in Figure 4A is unambiguously mediated by direct interactions between BoVapB1 and BoVapC1.

### BoVapC1-mediated cell elongation and its bacteriostatic effects

**FIGURE 5.**
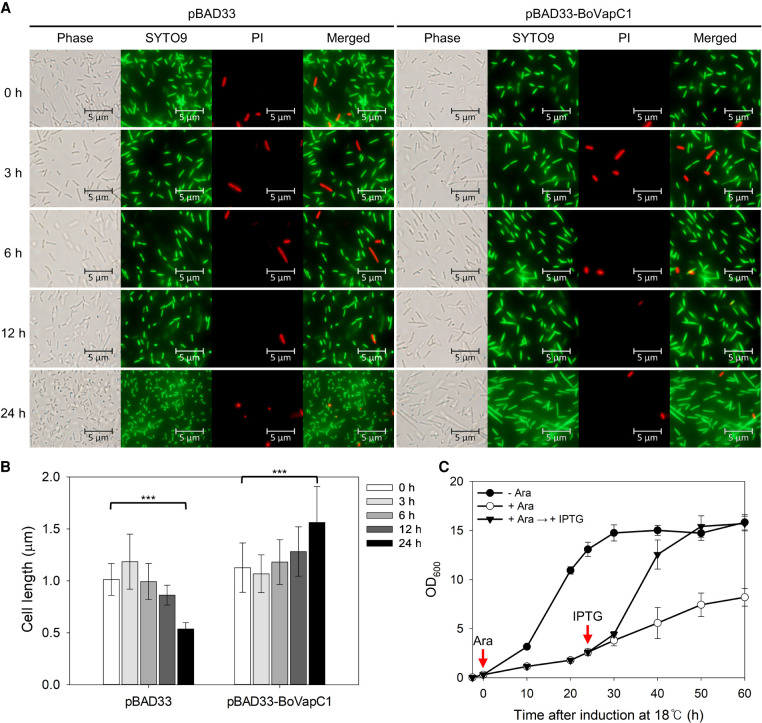
Bacteriostatic effect of BoVapC1 on *E. coli* growth. (*A*) Live/dead staining of BL21(DE3) cells expressing BoVapC1. *E. coli* BL21(DE3) transformants harboring pBAD33 or pBAD33-BoVapC1 were grown and stained for fluorescence microscopy as described in Materials and Methods. (*B*) Effect of BoVapC1 expression on the cell length of *E. coli*. Cell length data from Figure 5A was obtained using the MicrobeJ plugin for ImageJ (Ducret et al. 2016). One hundred and thirty seven cells were counted for each condition. Error bars represent SD. Statistical significance was derived using the two-tailed *t*-test. (***) *P* < 0.001. (*C*) Resumption of cell growth by antitoxin expression. BL21(DE3) cells harboring both pET21c-BoVapB1 and pBAD33-BoVapC1 were grown in M9 media containing Amp and Cm at 37°C to the early exponential phase. Then, the bacterial culture was diluted fivefold in fresh M9 media supplemented without (●) or with 0.4% arabinose, respectively, and further incubated at 18°C for 60 h (○). At 24 h after toxin induction, IPTG was added at a final concentration of 0.1 mM (▾). Red arrows indicate induction starting points. Three independent experiments were carried out, and error bars represent SD.

Under stress conditions, free toxins released from antitoxins trigger either reversible cell growth arrest or programmed cell death (Hayes 2003). To determine whether the toxic effect of BoVapC1 was bacteriostatic or bactericidal, live/dead staining was conducted using cells expressing BoVapC1. BL21(DE3) cells harboring pBAD33 or pBAD33-BoVapC1 were cultured, and harvested after induction with arabinose, as described in the Materials and Methods. Propidium iodine (PI), which can penetrate only dead cells through the damaged membrane, and fluoresces red, was used to stain dead cells (Stocks 2004). For live cell staining, SYTO9, which is a membrane-intercalating dye fluorescing green, was used (Stocks 2004). As shown in Figure 5A, upon induction by arabinose, both control and toxin-expressing cells were stained with SYTO9, but not with PI, indicating that most cells were alive. These findings were further confirmed using quantitative analysis to calculate the percentage of live cells, as described in the Materials and Methods. After the induction of BoVapC1 for 24 h, nearly all cells were found to be alive (Supplemental Fig. S7A). Of note, the control strain with pBAD33 exhibited reduced cell length after 24 h induction at 18°C (Fig. 5A,B), a finding which is consistent with that of a previous report showing shorter cells at lower temperature (Trueba et al. 1982). Conversely, upon the induction of BoVapC1, gradual elongation of cell length was observed (Fig. 5B). These findings suggest that although the growth-inhibitory effect of BoVapC1 blocked cell division, it was not lethal.

This bacteriostatic phenotype was further verified using cell growth resumption experiments. BL21(DE3) cells cotransformed with pBAD33-BoVapC1 and pET21c-BoVapB1 were grown to the early exponential phase at 37°C, then transferred to 18°C. After the induction of BoVapC1 with arabinose for 24 h at 18°C, cells were harvested and resuspended with M9 minimal medium containing 0.1 mM IPTG, followed by further incubation at 18°C up to 60 h. As shown in Figure 5C, upon the induction of BoVapB1, growth resumed in the BoVapC1-expressing cells. This result was also confirmed by spotting assays as described previously (Li et al. 2021). Cells expressing BoVapC1 for 24 h at 18°C were revived in a noninducing plate (Supplemental Fig. S7B), suggesting that the effect of BoVapC1 is bacteriostatic rather than bactericidal.

### tRNA^fMet^ cleavage activity of BoVapC1 in *E. coli*

**FIGURE 6.**
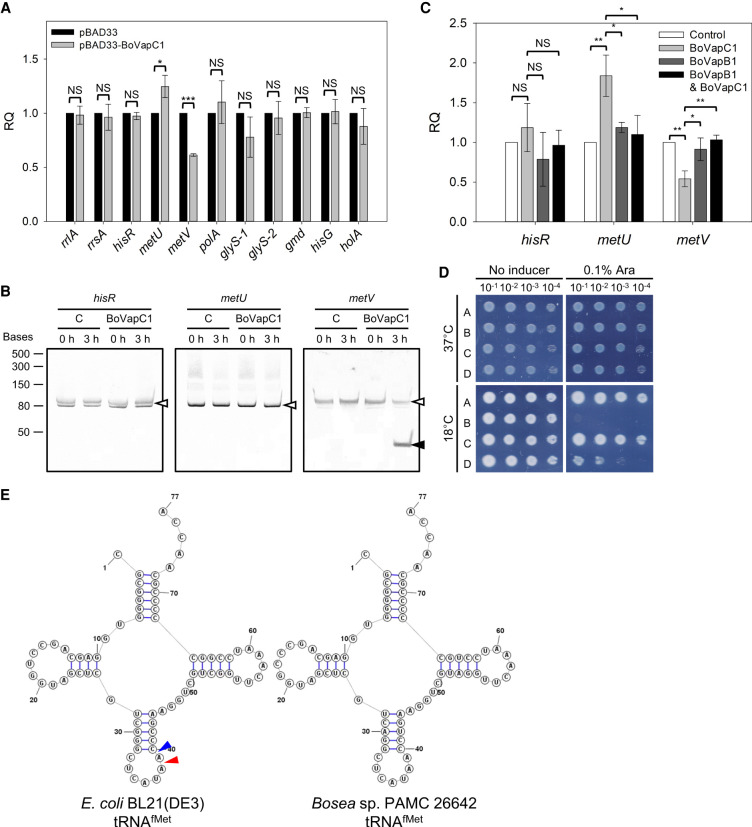
tRNA^fMet^-specific cleavage activity of BoVapC1. (*A*) qRT-PCR analyses of the level of mRNAs, rRNAs, and tRNAs. BL21(DE3) cells harboring pBAD33 or pBAD33-BoVapC1 were cultivated as described in Materials and Methods. Total RNAs extracted from these cells were utilized for qRT-PCR analyses. Experiments were independently conducted three times, and error bars represent SD. NS, nonsignificant; (*) *P* < 0.05; (***) *P* < 0.001. (*B*) Northern blotting analyses of tRNAs. RNAs were extracted from BL21(DE3) cells carrying pBAD33 (*C*) or pBAD33-BoVapC1 (BoVapC1) before (0 h) and after induction (3 h) and blotted with biotin-labeled oligonucleotides which are specific to the tRNAs as described in Materials and Methods. Full-length or cleaved tRNAs are indicated with open or filled arrowheads, respectively. Low Range ssRNA Ladder (New England Biolabs) was used as a size marker. (*C*) Inhibition of BoVapC1 ribonuclease activity by BoVapB1. BL21(DE3) cells harboring plasmid pairs were cultivated as described in Materials and Methods. Total RNAs extracted from these cells were utilized for qRT-PCR analyses. Experiments were independently performed three times. Error bars represent SD. NS, nonsignificant; (*) *P* < 0.05; (**) *P* < 0.01. (*D*) Growth restoration by overexpression of *metZWV* in BoVapC1-expressing cell. The BL21(DE3) cells pretransformed with pBAD33 or pBAD33-BoVapC1 were double transformed with pBR322 or pBR322-metZWV. The spotting assay was conducted as described in Figure 2. A; cells with pBAD33/pBR322, B; pBAD33-BoVapC1/pBR322, C; pBAD33/pBR322-metZWV, and D; pBAD33-BoVapC1/pBR322-metZWV. (*E*) The sequences and secondary structures of tRNA^fMet^s. The structures of tRNA^fMet^ of *E. coli* BL21(DE3) and *Bosea* sp. PAMC 26642 were drawn using StructureEditor (Reuter and Mathews 2010). Blue and red arrows indicate the positions cleaved by *S. flexneri* VapC (Winther and Gerdes 2011) and NTHi VapC (Walling and Butler 2018), respectively.

In various bacteria, VapC toxins inhibit protein synthesis through the hydrolytic cleavage of different RNAs, resulting in cell growth arrest (Ramage et al. 2009; Winther and Gerdes 2009). VapC homologs from *S. flexneri*, *Salmonella enterica*, nontypeable *Haemophilus influenzae* (NTHi), *Leptospira interrogans*, and a large number of VapCs from *M. tuberculosis* cleave tRNA site-specifically, between the anticodon stem and loop (Winther and Gerdes 2011; Lopes et al. 2014; Winther et al. 2016; Walling and Butler 2018). VapC20 and VapC26 from *M. tuberculosis* target and cleave the sarcin-ricin loop of 23S rRNA (Winther et al. 2013, 2016). VapC homologs from *M. smegmatis* and *S. solfataricus* cleave mRNAs in a sequence-specific manner (Maezato et al. 2011; McKenzie et al. 2012). Therefore, to examine whether BoVapC1 also has RNA cleavage activity and to identify the RNA substrate(s) for BoVapC1, we conducted qRT-PCR. Total RNAs were extracted from BL21(DE3) cells harboring pBAD33 or pBAD33-BoVapC1 at 3 h after induction and cDNAs were then synthesized. qRT-PCR was performed using cDNAs and primer sets relevant to two rRNAs, including *rrlA* (23S rRNA) and *rrsA* (16S rRNA), five mRNAs of various sizes (*polA*; ∼3 kb, *glyS*; ∼2 kb, *gmd*, *hisG*, and *holA*; ∼1 kb), and three tRNAs (the sole histidine tRNA; *hisR*, the elongator methionine tRNA; *metU*, and the initiator methionine tRNA; *metV*) (Supplemental Table S1). As shown in Figure 6A, there was no significant change in the amounts of two rRNAs and these five mRNAs. In contrast, the transcript level of *metV* decreased 1.63-fold by BoVapC1 expression, while that of *metU* increased by 1.25-fold, and that of *hisR* was not affected in BoVapC1-expressing cells (Fig. 6A). To further verify the tRNA^fMet^-specific cleavage activity of BoVapC1, northern blotting was carried out using RNAs extracted from BL21(DE3) cells harboring pBAD33 or pBAD33-BoVapC1 at 0 h and 3 h after induction. The full-length tRNA^fMet^ (77 nt) was observed intact in BL21(DE3) cells carrying pBAD33 regardless of induction (Fig. 6B, left two lanes, right panel). In contrast, after BoVapC1 induction, cleaved tRNA^fMet^ (<50 nt), which was not detected before induction, appeared with a concomitant reduction of full-length tRNA^fMet^ (Fig. 6B, right two lanes, right panel). The elongator tRNAs, tRNA^His^ and tRNA^Met^, remained constant without any degradation even after BoVapC1 overexpression (Fig. 6B, left and middle panels). Consistent with colony formation phenotype, no notable change was observed in those RNAs at 37°C (Supplemental Fig. S8). These results suggest that BoVapC1 specifically cleaves the initiator tRNA^fMet^ only at low temperatures with a site-specific endoribonuclease activity. The decline in the level of tRNA^fMet^ caused by BoVapC1 expression was neutralized by the expression of BoVapB1 antitoxin (Fig. 6C). The increased level of *metU* in BoVapC1-expressing cells also reverted to the normal level by coexpression of BoVapB1. No significant change was noted in the level of *hisR* by the expression of BoVapB1, BoVapC1, or both proteins.

These results led us to examine whether the overexpression of *metZWV* operon encoding tRNA^fMet^ could attenuate the BoVapC1-mediated growth inhibition at 18°C. Thus, we cloned *metZWV* operon into the pBR322 vector. BL21(DE3) cells harboring plasmid pairs (pBAD33/pBR322, pBAD33-BoVapC1/pBR322, pBAD33/pBR322-metZWV, or pBAD33-BoVapC1/pBR322-metZWV) were prepared and spotted on M9 agar plates with or without 0.1% arabinose. As shown in Figure 6D, overexpression of *metZWV* partially restored the growth defect caused by BoVapC overexpression at 18°C.

Our results indicate that initiator tRNA^fMet^ of *E. coli* can serve as a substrate for the toxin BoVapC1 at 18°C, which causes inhibition of translation, and subsequently growth arrest, and that BoVapB1 neutralized BoVapC1 by inhibiting the ribonuclease activity of BoVapC1, resulting in growth resumption. Furthermore, given the high sequence identity between *E. coli* and *Bosea* sp. PAMC 26642 tRNA^fMet^s (Fig. 6E), BoVapC1 likely controls cell growth of *Bosea* sp. PAMC 26642 at low temperature using the same mechanism.

### Contribution of BoVapBC1 to cold tolerance

**FIGURE 7.**
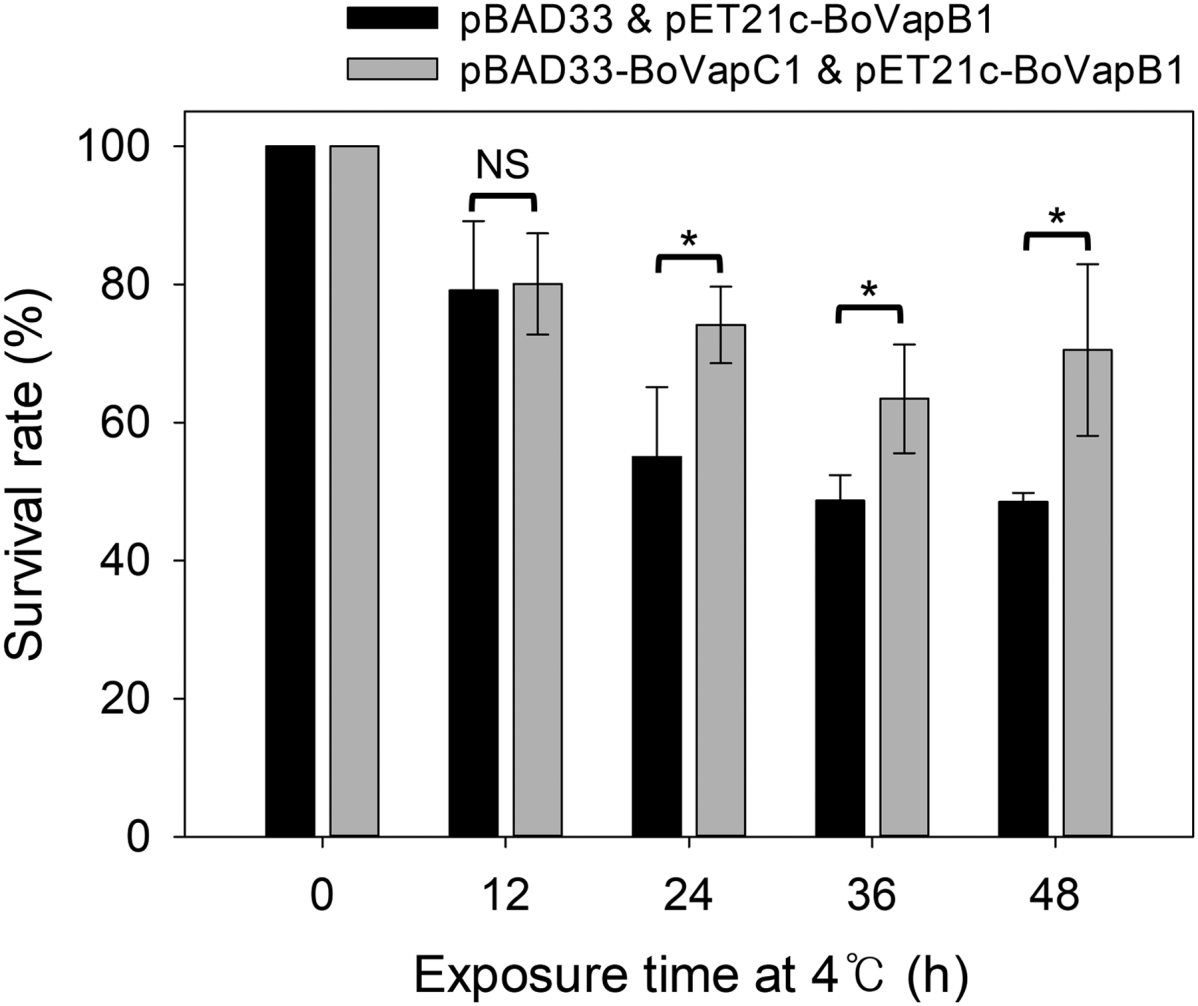
Survival rates of cells expressing BoVapC1 after exposure to 4°C. The survival rates were obtained by calculating the percentage of the number of CFU in the BoVapC1-induced cells, compared with those in control cells. Experiments were performed in three independent repetitions and error bars represent SD. Statistical significance was derived from the two-tailed *t*-test. NS, nonsignificant; (*) *P* < 0.05.

As mentioned earlier, TA systems were found to be lacking in other *Bosea* species occurring in a comparatively mild temperature environment (Supplemental Table S3). Of the eight *Bosea* species with whole genome sequence available, four do not have TA systems, and *B. vestrisii* and *B. caraganae* have two TA loci each. Interestingly, *B. psychrotolerans,* a psychrotrophic bacterium that can grow at 5°C (Albert et al. 2019), has five TA modules, suggesting that TA systems in *Bosea* sp. PAMC 26642 are important for its cold tolerance. Therefore, to investigate whether the BoVapBC1 showing low temperature-specific activity is involved in cold tolerance, we compared the survival rates of BL21(DE3) cells with pBAD33/pET21c-BoVapB1 or pBAD33-BoVapC1/pET21c-BoVapB1. After induction with 0.4% arabinose for 3 h at 18°C, the cultures were moved to refrigeration temperature (4°C), followed by static incubation for a further 48 h. The cells were harvested at 12-h intervals and the survival rates were analyzed as described in Materials and Methods. As the exposure time increased, the survival rates of cells harboring pBAD33 gradually reduced and reached 48.51% at 48 h (Fig. 7). The minimum survival rate of BoVapC1-expressing cells was 63.43% at 36 h; nevertheless, the survivability was higher than that of the control strain throughout the incubation. This finding suggests that BoVapBC1 enhanced the survivability of *E. coli* at 4°C and further implies that it plays an important role in cold tolerance of *Bosea* sp. PAMC 26642.

## DISCUSSION

In this study, we found 12 putative type II TA systems in *Bosea* sp. PAMC 26642 isolated from Arctic lichens. Five *vapBC* loci, four *higBA* loci, one *relBE* locus, one *hicAB* locus, and one *ficAT* locus are encoded in the genome of *Bosea* sp. PAMC 26642 (Fig. 1). Of the 12 predicted toxins, nine toxins were found to have toxic effects on the growth of *E. coli*, which were recovered by coexpression of their cognate antitoxins (Fig. 2). Our studies focused on the BoVapBC1 (AXW83_01400-AXW83_01405) TA module because BoVapC1, which has a high sequence similarity to other VapCs (Fig. 3A), was the only toxin among the nine functional toxins whose growth-inhibitory activity was inducible by low temperatures (Fig. 2).

Even though many VapC family toxins target tRNA, rRNA, or mRNA, most of them are reported to cleave tRNAs. Of the thirteen VapC toxins in *M. tuberculosis*, eleven VapCs target elongator tRNAs, and the remaining two VapCs, VapC20 and VapC26, cleave 23S rRNA (Winther et al. 2013, 2016; Cruz et al. 2015). In addition, VapC homologs in *S. enterica*, *S. flexneri*, *L. interrogans*, and NTHi specifically cleave tRNA^fMet^ in a sequence-specific and stem–loop structure-specific manner (Winther and Gerdes 2011; Lopes et al. 2014; Walling and Butler 2018). However, VapC homologs of these four mesophilic bacteria have not been explored for temperature-dependent functionality in *E. coli*. Nevertheless, these VapC homologs of *S. enterica*, *S. flexneri*, and NTHi have been implicated in bacteriostatic dormancy (Winther and Gerdes 2011; Ren et al. 2012), like we have shown that BoVapC1 induction had a bacteriostatic effect on *E. coli* growth with elongated morphologies (Fig. 5). Similarly, in the view of tRNA inactivation, acetylation of tRNA by TacT and phosphorylation of glutamyl-tRNA-synthetase by HipA caused disturbances in translation initiation or elongation and ultimately arrested cell growth or division rather than cell death (Korch and Hill 2006; Helaine et al. 2014; Cheverton et al. 2016; Rycroft et al. 2018; Walling and Butler 2019).

Our qRT-PCR and northern blotting analyses indicated that the levels of the full-length tRNA^fMet^ were significantly reduced by the expression of BoVapC1 in *E. coli* only at low temperature (Fig. 6). In *E. coli*, initiator tRNA^fMet^s are produced from the *metZWV* and *metY-nusA-infB* operons (Ishii et al. 1984; Kenri et al. 1994). The *metZWV* operon contributes to ∼75% of the total intracellular tRNA^fMet^, and the remaining were produced from *metY* (Kenri et al. 1994). The deletion or reduced expression of *metZWV* conferred a cold-sensitive phenotype, which was not present in *metY* deletion, suggesting that the total cellular level of tRNA^fMet^ is crucial for growth at low temperature (Kapoor et al. 2011). After cold shock, the expression of *metY* as well as *infB* encoding translation initiation factor 2 (IF2) are induced from the *metY-nusA-infB* operon (Brandi et al. 2019). The levels of the other two translation initiation factors, IF1 and IF3, are also increased upon temperature downshift (Giangrossi et al. 2007; Giuliodori et al. 2007), indicating that translation initiation is critical for adjustment to cold growth conditions. The tRNA^fMet^ of *Bosea* sp. PAMC 26642 has 94% nucleotide sequence identity with that of *E. coli*. Particularly, the anticodon stem–loop region, which is the target site of the VapC homologs (Winther and Gerdes 2011; Lopes et al. 2014), of *Bosea* sp. PAMC 26642 tRNA^fMet^ is identical to that of *E. coli* BL21(DE3) (Fig. 6E), suggesting that BoVapC1 is likely to cleave the tRNA^fMet^ in the *Bosea* sp. PAMC 26642 cells through the same cleavage mechanism. Unlike *E. coli*, *Bosea* sp. PAMC 26642 encodes only a single copy of the initiator tRNA^fMet^ gene in its genome. Therefore, it is likely that BoVapC1 expression in *Bosea* sp. PAMC 26642 effectively triggers the reduction in the level of tRNA^fMet^, which inhibits the formation of the translation initiation complex, consequently leading to the growth arrest at low temperature.

Taking the results of this study into account, the increased toxicity observed at 18°C compared with 37°C, is possibly associated with an increased need for a tRNA^fMet^ target at a temperature that is this low; however, why tRNA^fMet^ was cleaved by BoVapC1 only at 18°C (Fig. 6; Supplemental Fig. S8) is still unclear. Several type II toxins have been reported to be functionally affected by temperature, in the same manner as BoVapC1. In *Pseudomonas putida*, deletion of the *graA* antitoxin gene causes a reduction in the colony formation rate, which becomes apparent at low temperatures (Tamman et al. 2014). This reduction was caused by the decreased activity of GraT at 37°C, not by the low expression level of *graA* (Tamman et al. 2014). In the case of the *E. coli* YafQ/DinJ TA system, either the deletion of the *dinJ* antitoxin gene or the overexpression of YafQ in the *yafQ*/*dinJ* operon-deleted cell leads to significant reduction in cell metabolism and proliferation at lower temperatures (Zhao et al. 2016). The lytic phenotype generated by the overexpression of another *E. coli* toxin, EzeT, becomes more pronounced at temperatures below 30°C (Rocker and Meinhart 2015). Another zeta toxin, Ld_ζ1 domain of *Leishmania donovani*, also showed pronounced growth-inhibitory activity at lower temperatures (Srivastava et al. 2019).

Considering the temperature-sensitive phenotypes observed in our study and several other TA systems, we can propose three hypotheses to explain why BoVapC1 was active only at 18°C as follows: (i) BoVapC1 may aim to the target(s) such as tRNA^fMet^ that plays an important role during cold adaptation, (ii) the activity of BoVapC1 may be controlled by conformational changes induced by temperature, or (iii) there may be another regulator protein inactivating BoVapC1 at 37°C or activating it at 18°C.

Cold-adapted proteins of psychrophilic bacteria have evolved to increase structural flexibility as well as stability at low temperature, whereas they show low thermal stability and heat labile-activity at higher temperatures (Feller 2013; Bruno et al. 2019). Therefore, as shown in Supplemental Figure S8, it is likely that the stability and/or activity of BoVapC1 diminishes at 37°C with no tRNA^fMet^ cleavage activity, and that the conformation at 18°C gains access and catalytic activity to the substrate. Nevertheless, the absence of BoVapC1 activity at 37°C does not appear to be a result of either reduced expression or instability of the *BovapC1* mRNAs owing to the fact that a higher transcript level of *BovapC1* was noted at 37°C compared with that at 18°C (Supplemental Fig. S9). Furthermore, we cannot rule out the possibility that a trans- or cross-regulator may modulate the function of BoVapC1 at either temperature (Yang et al. 2010; Zhu et al. 2010; Otsuka and Yonesaki 2012).

Interestingly, TA systems are more abundant in bacteria living in stressful environments compared with those living in constant environments, implying that TA modules function as cellular stress-response elements (Pandey and Gerdes 2005). Recent studies have demonstrated that the VapBC module, which is the most abundant TA subfamily in type II (Arcus et al. 2011), is indeed associated with adaptation to environmental stress through persister cell and biofilm formation (Ren et al. 2012; Levin-Reisman et al. 2017; Eroshenko et al. 2020). The VapBC-1 in NTHi contributes to survival capability under nutrient deficient condition or during infection process (Cline et al. 2012; Ren et al. 2012). VapC4, VapC20, VapC22, VapC26, and VapC45 of *M. tuberculosis* VapBC modules are associated with enhanced survival in macrophages or against oxidative stress (Rengarajan et al. 2005; Agarwal et al. 2020). In addition, the heat-shock inducible VapBC system of *S. solfataricus* increases viability under thermal stress conditions (Cooper et al. 2009; Maezato et al. 2011). Nevertheless, the functional relationship between survival under cold stress conditions and VapBC system has not been elucidated yet. Therefore, even though our findings are based on experiments carried out in *E. coli*, it is of a great interest how, compared to other *Bosea* species in a mild habitat, *Bosea* sp. PAMC 26642 has evolved this system to endure the extremely low temperatures in the Arctic.

In this study, we identified nine functional type II TA systems in the *Bosea* sp. PAMC 26642 and characterized the possible function of the BoVapBC1 system at low temperature. BoVapC1, which cleaved tRNA^fMet^ in a low temperature-dependent manner, enhanced the survivability of *E. coli* after being exposed to refrigeration temperature (Fig. 7). Presumably, this is the first study to identify temperature-dependent VapC toxin; however, further in vivo and structural studies are required to understand the role and mechanism of the BoVapBC TA system in the adaptation to environment in this extremophile.

## MATERIALS AND METHODS

### Bacterial strains, media, and growth conditions

The *E. coli* BL21(DE3) strain was grown at 37°C or 18°C in Luria–Bertani (LB) or M9 minimal media supplemented with 0.2% casamino acids and 0.4% glucose. When necessary, antibiotics were added at the following concentrations: ampicillin (Amp); 100 µg/mL and chloramphenicol (Cm); 50 µg/mL.

### Plasmid construction

Twelve putative type II toxin and antitoxin genes in *Bosea* sp. PAMC 26642 were identified using TAfinder (https://bioinfo-mml.sjtu.edu.cn/TAfinder/TAfinder.php), and the open reading frames of those toxins were synthesized and cloned in pUCIDT (Cosmogenetech), yielding pUCIDT-toxin plasmids (Supplemental Table S2). The pUCIDT-toxin plasmids were digested by the appropriate restriction enzymes. The digested DNA fragments carrying toxin genes were cloned into the *Nde*I-*Hin*dIII or *Nde*I-*Sac*I sites of the pBAD33 vector containing arabinose-inducible *araBAD* promoter, and we used LB medium supplemented with 0.2% glucose to strongly repress the promoter (Guzman et al. 1995). Nine antitoxin genes were also synthesized and cloned into the *Nde*I-*Hin*dIII sites of the pET21c vector containing β-D-1-thiogalactopyranoside (IPTG)-inducible T7 promoter, yielding pET21c-antitoxin plasmids.

Point mutations were introduced into BoVapB1 or BoVapC1 by site-directed mutagenesis PCR using pUCIDT-BoVapB1 or pET21c-BoVapC1 as templates, respectively, and the primer sets listed in Supplemental Table S1. The toxin DNA fragments carrying each mutation were ligated into pBAD33, yielding pBAD33-BoVapC1_D7A_, pBAD33-BoVapC1_E42A_, pBAD33-BoVapC1_D98A_, and pBAD33-BoVapC1_E119A_. The mutated BoVapB1 DNA fragments were ligated into pET21c, yielding pET21c-BoVapB1_W53R_, pET21c-BoVapB1_F56SF57S_, and pET21c-BoVapB1_W53RF56SF57S._ To clone the *metZWV* operon, the PCR-amplified DNA fragments were ligated with the *Sma*I site of pBR322, yielding pBR322-metZWV. All plasmids and primers used in this study are listed in Supplemental Tables S1, S2.

### In vivo toxin–antitoxin neutralization test

To determine the minimal concentration of arabinose for the neutralization assay, BL21(DE3) cells were transformed with pBAD33 or pBAD33-toxin and plated on M9 agar plates containing Cm, followed by incubation at 37°C overnight. Then, each transformant was scraped from the agar plates, and resuspended into 5 mL of M9 minimal media. The resuspension was diluted in the same media to an optical density at 600 nm (OD_600_) of 0.2, and serially diluted 10^−1^, 10^−2^, 10^−3^, and 10^−4^-fold. A total of 2 µL of each dilution was spotted on M9 agar plates containing Cm with various concentrations of arabinose, or without inducer. To assess the effect of antitoxin expression on *E. coli* growth, serial dilutions of BL21(DE3) cells harboring pET21c or pET21c-antitoxin were prepared as described above and spotted on M9 agar plates containing Amp with or without IPTG.

For the neutralization assay, the serially diluted samples of BL21(DE3) harboring plasmid pairs (pBAD33/pET21c, pBAD33-toxin/pET21c, pBAD33/pET21c-antitoxin, or pBAD33-toxin/pET21c-antitoxin) were prepared and spotted on M9 agar plates containing Amp and Cm with or without inducers. For the neutralization assay for the BoVapBC1 TA system in liquid medium, overnight cultures of BL21(DE3) transformants with plasmid pairs (pBAD33/pET21c, pBAD33-BoVapC1/pET21c, pBAD33/pET21c-BoVapB1, or pBAD33-BoVapC1/pET21c-BoVapB1) were diluted 100-fold in M9 minimal media supplemented with Amp and Cm. After incubation at 37°C to the early exponential phase, the cultures were diluted fivefold with the same medium, and then inducers were added as follows: 0.4% arabinose, 0.1 mM IPTG, or both. The diluted cultures were incubated at 18°C for 24 h and continuously diluted fivefold every 6 h in fresh M9 minimal medium with or without inducers, and OD_600_ was measured.

### Live/dead cell staining for microscopy

Overnight cultures of BL21(DE3) cells harboring pBAD33 or pBAD33-BoVapC1 were diluted 100-fold in M9 minimal media containing Cm at 37°C, followed by cultivation to the early exponential phase. The cell cultures were diluted fivefold in fresh M9 media supplemented with Cm and 0.4% arabinose and incubated at 18°C for 24 h. A total of 1.5 mL of cell cultures at the OD_600_ of 0.8 were harvested by centrifugation at each point in time (0, 3, 6, 12, and 24 h) and washed twice with 1.2 mL of 0.85% NaCl. The pellets were resuspended in 1.2 mL of 0.85% NaCl. Then, the samples were stained using LIVE/DEAD BacLight Bacterial Viability Kit (Thermo Fisher Scientific), following the manufacturer's instructions. Briefly, 12 µL of the diluted dye mixture of SYTO9 and PI was added to each bacterial suspension. After incubation at room temperature in the dark for 15 min, the bacterial suspensions were concentrated 10-fold. A total of 5 µL of the stained bacterial suspension was placed between the slide and cover glasses, and cells were observed with an Axio Observer (Zeiss) at 1000× magnification.

To quantitate each staining, the bacterial suspensions were diluted to an OD_600_ of 0.2 in 1 mL with 0.85% NaCl. Equal volumes of SYTO9 and PI dyes were mixed beforehand, following which 3 µL of dye mixture was added to each bacterial suspension. The stained samples were incubated at room temperature in the dark for 15 min and transferred to a cuvette. Fluorescence intensity was determined with a fluorescence spectrophotometer (Hitachi F-7000) at 470 nm for excitation and wavelength spectrums of 510–540 nm (for SYTO9) and 620–650 nm (for PI) for emission. The percentage of live bacteria was calculated by dividing the SYTO9 intensity into the sum of SYTO9 and PI intensities.

### Growth resumption test

Overnight cultures of BL21(DE3) cells cotransformed with pET21c-BoVapB1 and pBAD33-BoVapC1 were diluted 100-fold in M9 media supplemented with Amp and Cm and incubated at 37°C to the early exponential phase. Then, the cultures were diluted fivefold into fresh M9 media with or without 0.4% arabinose, and further incubated at 18°C for 60 h. At 24 h after arabinose addition, the cells were harvested by centrifugation and resuspended in the same volume of M9 minimal medium supplemented with 0.1 mM IPTG. OD_600_ was measured at every 10 h.

### Quantitative real time PCR (qRT-PCR) analysis

BL21(DE3) cells harboring pBAD33 or pBAD33-BoVapC1, grown to the early exponential phase, were diluted fivefold in M9 minimal media supplemented with Cm and 0.4% arabinose and incubated at 37°C or 18°C for 3 h. For the neutralized cells, BL21(DE3) cells cotransformed with pBAD33/pET21c, pBAD33-BoVapC1/pET21c, pBAD33/pET21c-BoVapB1, or pBAD33-BoVapC1/pET21c-BoVapB1 were grown to the early exponential phase and diluted fivefold in M9 minimal media supplemented with Amp, Cm, 0.4% of arabinose, and 0.1 mM IPTG, followed by incubation at 18°C for 3 h. At the indicated time point, 2 mL of cell cultures for OD_600_ of 1.0 were harvested by centrifugation.

Total RNA was extracted using the hot phenol method with some modification (Sarmientos et al. 1983). BL21(DE3) cells were grown to OD_600_ of 1.0, and 1.5 mL of culture was harvested by centrifugation at 13,000 rpm for 1 min. The cell pellets were resuspended in 500 µL of Solution A [20 mM sodium acetate, 10 mM ethylenediaminetetraacetic acid (EDTA), 0.5% sodium dodecyl sulfate (SDS)], and mixed with equal volumes of phenol (pH 4.3). After incubation at 60°C for 10 min and on ice for 5 min, centrifugation at 13,000 rpm was performed to remove the phenol. The supernatants were transferred to new tubes and mixed with 50 µL of 3 M sodium acetate (pH 5.2) and 1 mL of 100% cold ethanol. Following incubation at −20°C for 1 h, ethanol was removed by centrifugation at 13,000 rpm for 10 min at 4°C. The pellets were dissolved in Solution A without SDS and precipitated with ethanol once more. Ethanol was removed by centrifugation, following which the pellets were washed with 75% ethanol. Finally, RNAs were dissolved in 100 µL of diethylpyrocarbonate (DEPC)-treated water. The concentration and purity of the RNA were determined using a NanoDrop One^C^ Spectrophotometer (Thermo Fisher Scientific), and the quality of RNA was validated by agarose gel electrophoresis. DNA contamination was removed by treatment with recombinant DNase I (RNase-free) (TaKaRa) according to the manufacturer's protocol. Then, cDNA synthesis was carried out using BioFACT RT Kit and 1 µg of total RNA. The qRT-PCR reaction was carried out as previously described (Choi et al. 2020). The *recA* gene served as an endogenous reference, and relative quantification values were calculated using the comparative *C*_T_ method (Livak and Schmittgen 2001). The primers used for qRT-PCR analysis are listed in Supplemental Table S1. The level of tRNA was examined using primer sets to amplify the anticodon stem and loop regions.

### Northern blotting

A total of 20 µg of total RNAs, denatured with formamide by heating at 70°C for 10 min, were separated on 15% denaturing polyacrylamide gel containing 8 M urea in 1× TBE [45 mM Tris-borate (pH 8.3), 1 mM EDTA]. The gel was run at 100 V for ∼2.5 h until the bromophenol blue dye front reached to 0.5 cm above the bottom of the gel. Then, the RNAs were transferred to a nylon membrane (Hybond N+, Amersham) by electroblotting using Bio-Rad Semi-Dry electrophoretic transfer cell at constant 20 V for 1 h. After transfer, the membrane was cross-linked by exposure to ultraviolet light (254 nm) at 120 mJ/cm^2^ for 1 min using CL-1000 UV crosslinker (UVP Inc.). Next, the membrane was prehybridized with hybridization buffer [100 mM sodium phosphate (pH 7.2), 0.2 mM EDTA, 1% SDS, 1 mg/ml bovine serum albumin] with 50 µg/mL denatured salmon sperm DNA for 4 h at 65°C. Then, the membrane was incubated at 52°C overnight with hybridization buffer supplemented with 500 ng/mL probes labeled with biotin at both ends (Supplemental Table S1). The membrane was washed sequentially with wash buffer I [2× SSC, 0.1% SDS], with wash buffer II [1× SSC 0.1% SDS], and with 0.1× SSC at 42°C for 15 min each. The biotin signals were detected using a Biotin Chromogenic Detection Kit (Thermo Fisher Scientific) following the manufacturer's instructions.

### Survival rate measurements

BL21(DE3) cells harboring pET21c-BoVapB1 were transformed with pBAD33 or pBAD33-BoVapC1 and grown in M9 minimal medium with Amp and Cm at 37°C to the early exponential phase. The cultures were diluted fivefold in M9 minimal media supplemented with Amp, Cm, and 0.4% arabinose and further incubated at 18°C for 3 h. Then, the cells were incubated at 4°C for 48 h. At the indicated time points, the cells were diluted in M9 minimal medium to an OD_600_: 0.1. The resuspension was further diluted 10^−5^-fold and plated on M9 agar plates with 0.1 mM IPTG, followed by incubation at 37°C. Colony forming units (CFU) were counted, and the survival rates were expressed as the percentage of the number of colonies in the treated samples (3, 6, 12, and 24 h), compared with that of the untreated samples (0 h).

### Statistical analysis

Results are presented as the mean ± SD of three independent experiments. The data were analyzed by two-tailed *t-*tests. The statistical analyses were performed with the following significance levels: NS, nonsignificant; (*) *P* < 0.05; (**) *P* < 0.01; (***) *P* < 0.001.

## SUPPLEMENTAL MATERIAL

Supplemental material is available for this article.

## Supplementary Material

Supplemental Material
