# Supplementary material for: Identification and characterization of VapBC toxin–antitoxin system in Bosea sp. PAMC 26642 isolated from Arctic lichens
Source: RNA. 2021 Nov;27(11):1374–89. doi: 10.1261/rna.078786.121 (PMC8522696; doi:10.1261/rna.078786.121)
Supplement: Supplemental Material [file supp_27_11_1374__DC1.html]

Identification and characterization of VapBC toxin–antitoxin system in Bosea sp. PAMC 26642 isolated from Arctic lichens — Supplemental Material 

# Identification and characterization of VapBC toxin–antitoxin system in *Bosea* sp. PAMC 26642 isolated from Arctic lichens

## Supplemental Material

- Supplemental\_Figures.pdf
- Supplemental\_Methods\_Figure\_Legends.docx
- Supplemental\_Table\_S1.docx
- Supplemental\_Table\_S2.docx
- Supplemental\_Table\_S3.docx
