# Supplementary figures and images for: Identification and characterization of VapBC toxin–antitoxin system in Bosea sp. PAMC 26642 isolated from Arctic lichens
Source: RNA. 2021 Nov;27(11):1374–89. doi: 10.1261/rna.078786.121 (PMC8522696; doi:10.1261/rna.078786.121)

Figure S1.

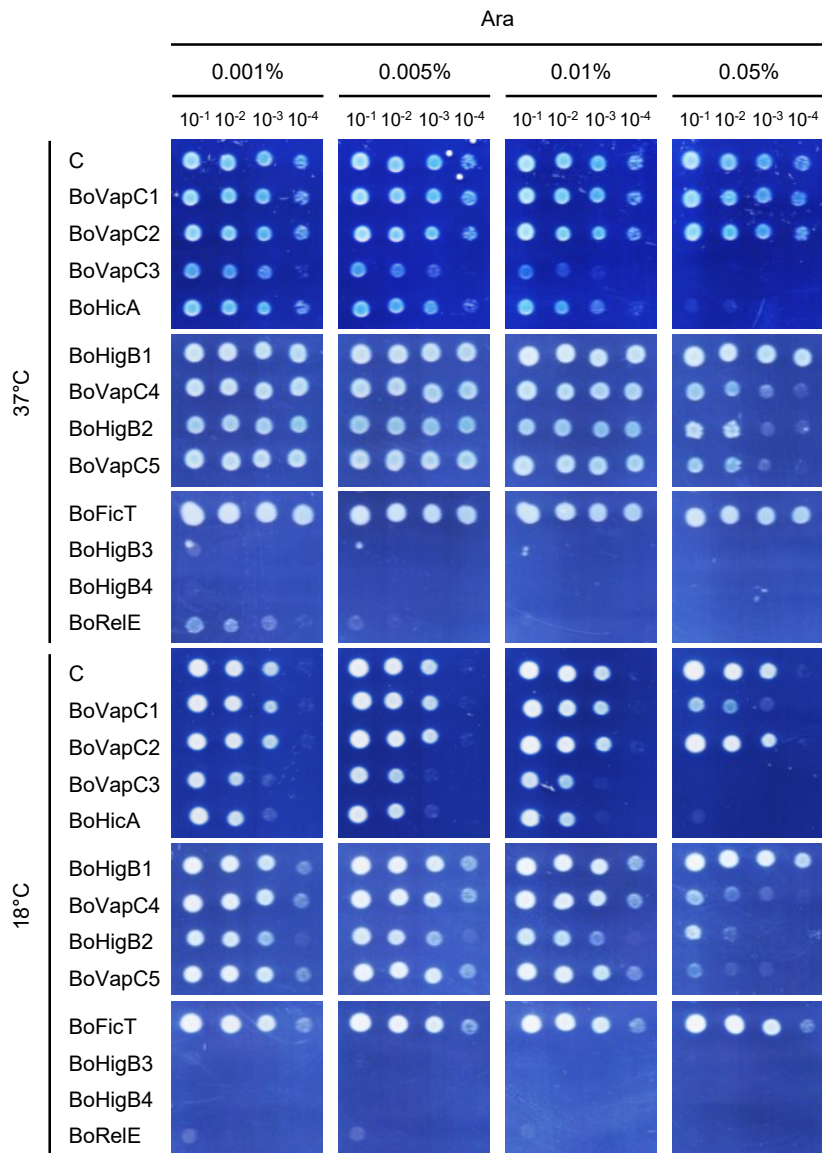

Figure S2.

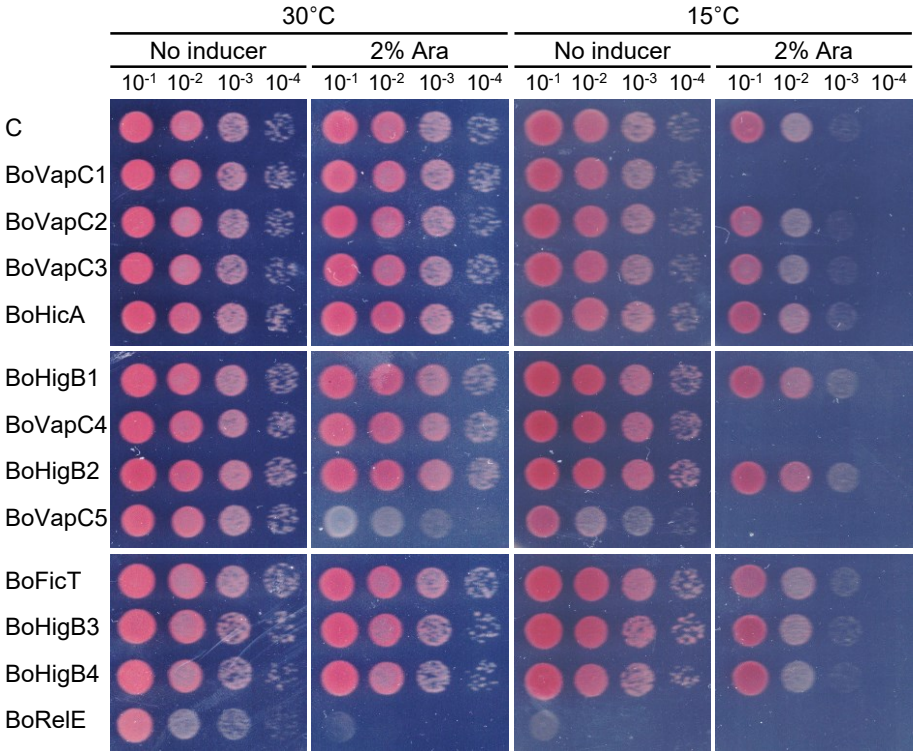

Figure S3.

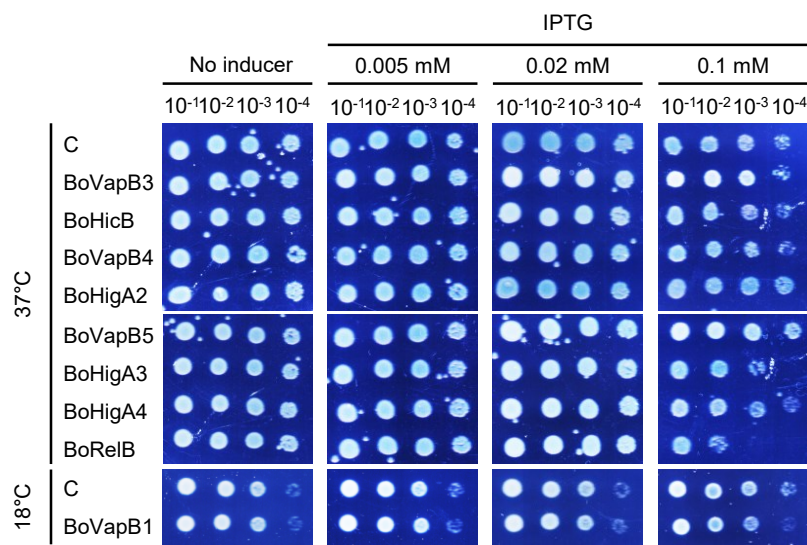

Figure S4.

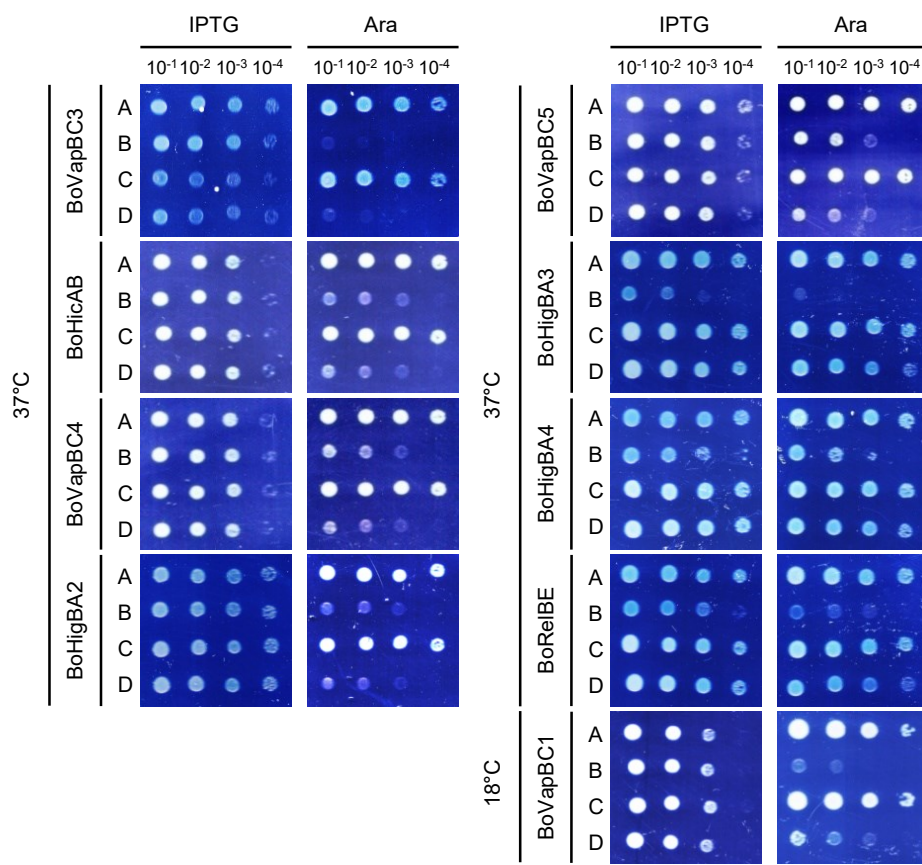

Figure S5.

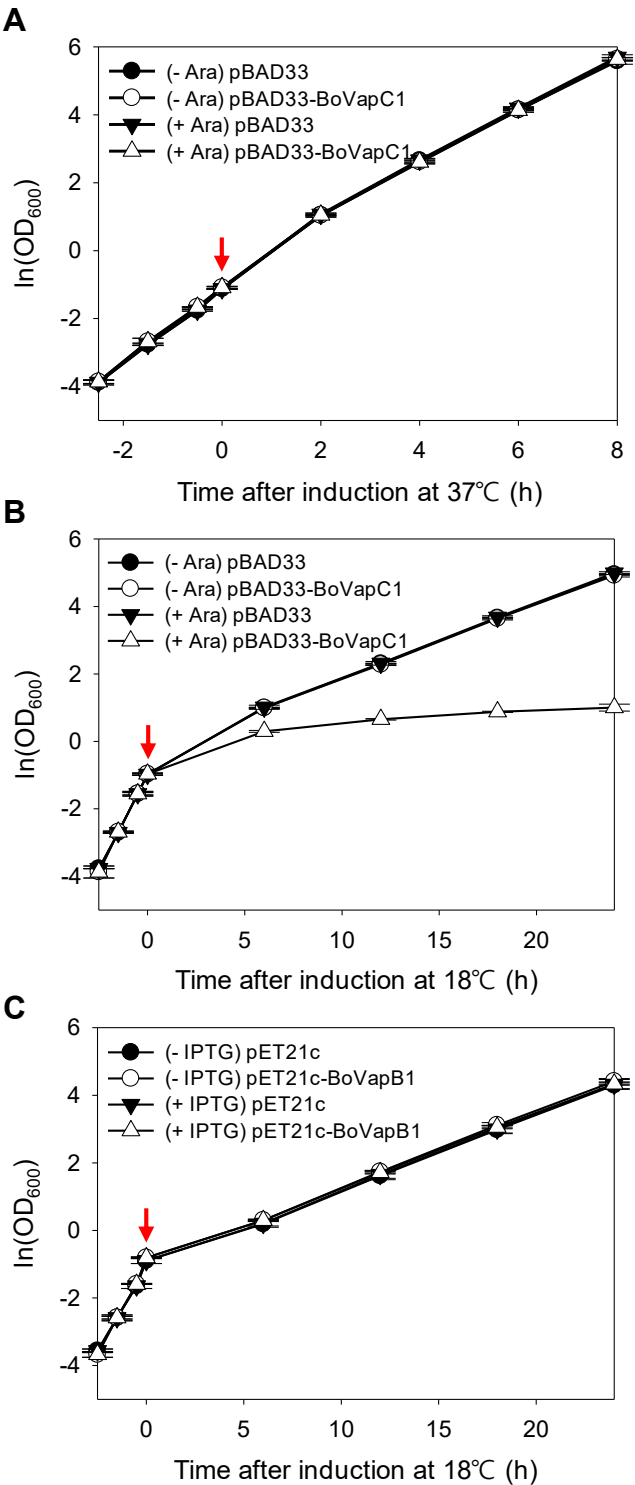

Figure S6.

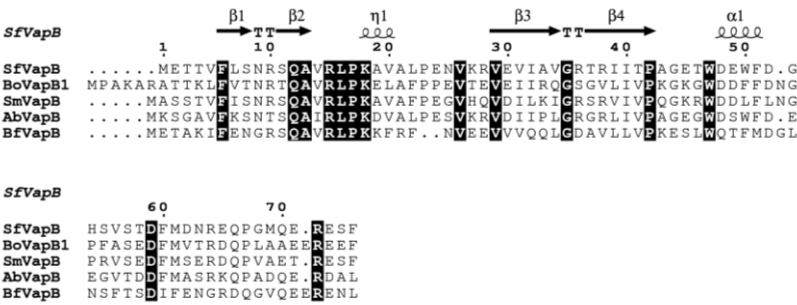

Figure S7.

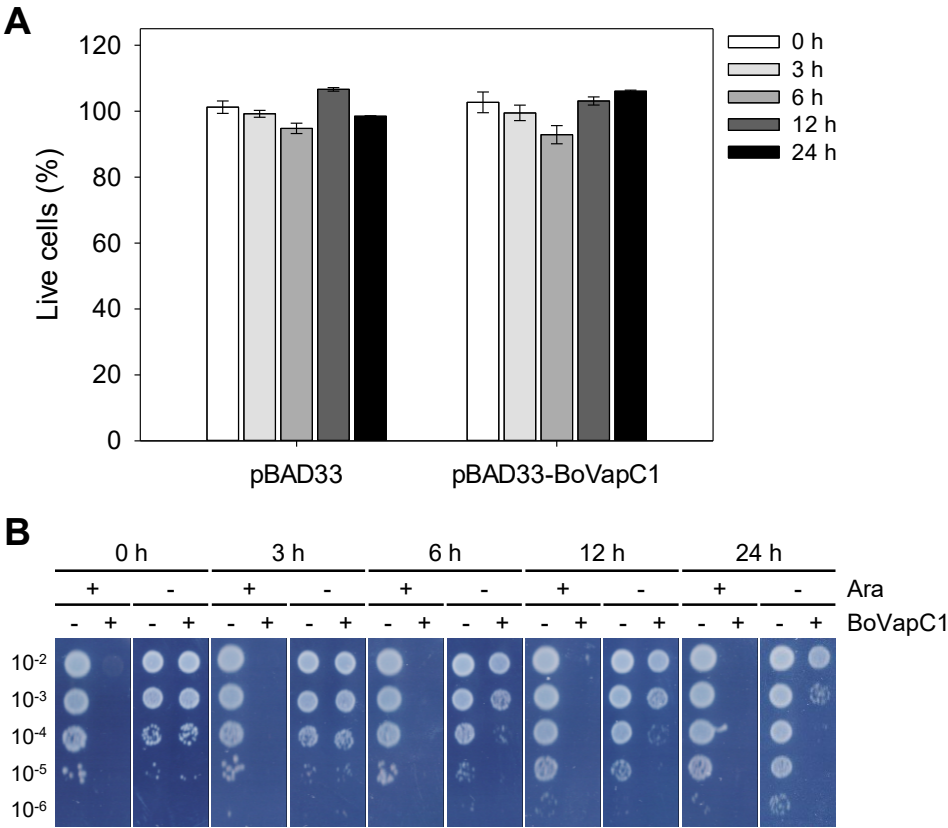

Figure S8.

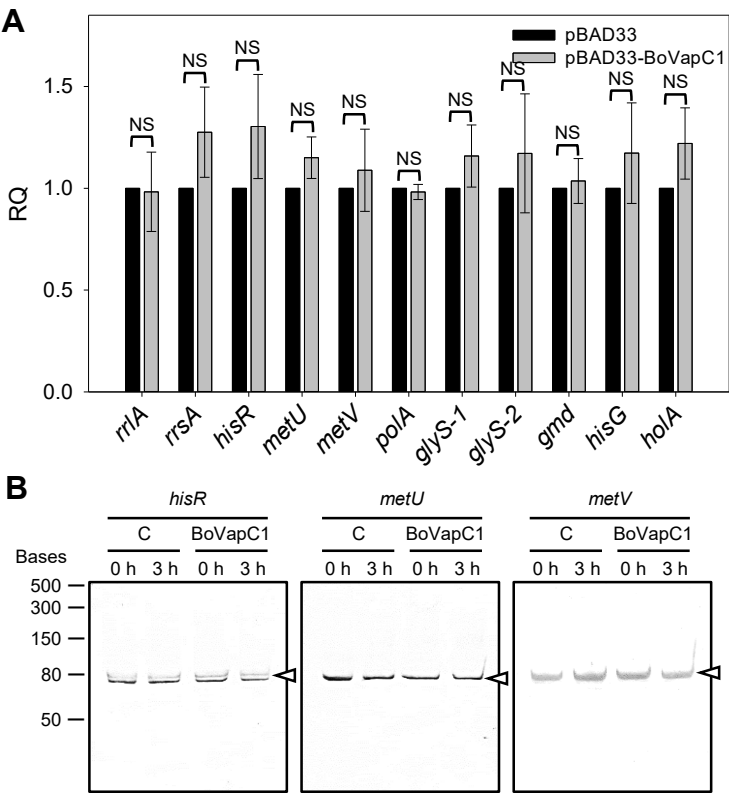

Figure S9.

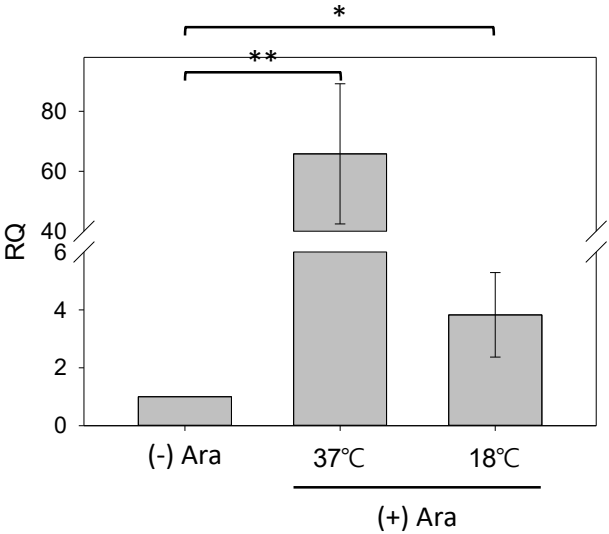

Supplement: Supplemental Material [file supp_078786.121_Supplemental_Figures.pdf]
