# Supplementary material for: Identification and characterization of VapBC toxin–antitoxin system in Bosea sp. PAMC 26642 isolated from Arctic lichens
Source: RNA. 2021 Nov;27(11):1374–89. doi: 10.1261/rna.078786.121 (PMC8522696; doi:10.1261/rna.078786.121)
Supplement: Supplemental Material [file supp_078786.121_Supplemental_Methods_Figure_Legends.docx]

**Supplemental Information**

**Identification and Characterization of VapBC Toxin-antitoxin System in *Bosea* sp.**

**PAMC 26642 Isolated from Arctic Lichens**

Hyerin Jeon^1,§^, Eunsil Choi^1,2,§^, and Jihwan Hwang^1,2*^

^1^Department of Microbiology, Pusan National University, Busan, Republic of Korea

^2^Microbiological Resource Research Institute, Pusan National University, Busan, Republic of Korea

**SUPPLEMENTAL METHODS**

**Toxicity test in *R. sphaeroides***

*R. sphaeroides* 2.4.1 cells were cultivated at 30°C in LB medium or Sistrom’s (SIS) medium (Sistrom 1962). When required, tetracycline (1 μg/ml) was added.

The arabinose-inducible expression vectors for *R. sphaeroides* 2.4.1 were constructed by ligating the *Nst*I-digested pBAD33 or pBAD33-toxin plasmids into the *Pst*I site of shuttle vector pRK415, yielding pRK-BAD or pRK-BAD-toxin (Supplemental Table S2). These plasmids were introduced into *R. sphaeroides* 2.4.1 through diparental conjugation mediated by *E. coli* S17-1. The 1 ml overnight cultures of S17-1 cells transformed with pRK-BAD or pRK-BAD-toxin plasmids and *R. sphaeroides* cells were harvested by centrifugation, washed twice with 1 ml of SIS medium, and resuspended in 30 μl of the same medium. The cell suspensions of donor and recipient were mixed and spotted on LB plate. After incubation at 30°C for 18 h, the mating spot was scrapped from the LB plate, washed with 1 ml of SIS medium, and resuspended in 500 μl of the same medium. 50 μl of the cell suspension was plated on SIS agar plate supplemented with Tc for the selection of conjugants.

For spotting assay, *R. sphaeroides* cells carrying pRK-BAD or pRK-BAD-toxin plasmids were grown aerobically in SIS medium with Tc at 30°C to OD_600_ of 0.4-0.5. The cultures were diluted in the same media to OD_600_ of 0.2, and serially diluted 10^−1^, 10^−2^, 10^−3^, and 10^−4^-fold. 3 μl of each dilution was spotted on LB agar plates containing Tc with or without 2% arabinose, followed by incubation at 30°C or 15°C.

**Supplemental Figure S1. The toxicity test of *Bosea* sp. PAMC 26642 toxins in *E. coli* growth.** *E. coli* BL21(DE3) cells transformed with pBAD33 or pBAD33-toxin were spotted on M9 agar plates supplemented with Cm and 0.001%, 0.005%, 0.01%, and 0.05% of arabinose, followed by incubation at 37℃ or 18℃. C indicates pBAD33.

**Supplemental Figure S2. The toxicity assay of *Bosea* sp. PAMC 26642 toxins in *R*. *sphaeroides*.** The cultures of *R. sphaeroides* cells harboring pRK-BAD or pRK-BAD-toxin were diluted and spotted on LB agar plates containing tetracycline with or without 2% arabinose, and the plates were incubated at 30℃ or 15℃. C indicates pRK-BAD.

**Supplemental Figure S3. The effect of antitoxins on *E. coli* growth.** *E. coli* BL21(DE3) cells were transformed with pET21c or pET21c-antitoxin. The transformant colonies were scraped and diluted as described in Fig. 2. Bacterial suspensions were diluted with M9 minimal media containing Amp. 2 µl of each suspension was spotted on M9 agar plate supplemented with Amp and 0 mM, 0.005 mM, 0.02 mM, and 0.1 mM IPTG. C indicates pET21c.

**Supplemental Figure S4. Individual induction of toxin or antitoxin in double transformant.** *E. coli* BL21(DE3) transformants in Fig. 2B cells were spotted on M9 agar plate supplemented with either arabinose or IPTG, and the plates were incubated at 37℃ or 18℃. A; cells with pBAD33/pET21c, B; pBAD33-toxin/pET21c, C; pBAD33/pET21c-antitoxin, and D; pBAD33-toxin/pET21c-antitoxin.

**Supplemental Figure S5. Growth inhibition by BoVapC1 and its recovery by BoVapB1 in liquid medium.** Assessment of growth inhibition effect of BoVapC1 in liquid media at 37℃ **(A)** or 18℃ **(B)**. *E. coli* BL21(DE3) cells harboring pBAD33 or pBAD33-BoVapC1 were grown at 37℃ in M9 media containing Cm to an early exponential phase. Then, the cultures were diluted five-fold with fresh M9 media supplemented with Cm and 0.4% arabinose and shifted at 37℃ or 18℃. During cultivation at these temperatures, the cultures were repeatedly diluted five-fold at every 2 h or 6 h, respectively, and their OD_600_ values were measured at the indicated time points. **(C)** Growth curves of *E. coli* BL21(DE3) expressing BoVapB1. *E. coli* BL21(DE3) cells harboring pET21c or pET21c-BoVapB1 were grown at 18℃ in M9 media containing Amp. At 0 h, 0.1 mM IPTG was added for expression of antitoxin. The cells were grown for 24 h at 18℃ after induction, and OD_600_ values were measured. Red arrows indicate induction starting points. Three independent experiments were carried out, and error bars represent SD.

**Supplemental Figure S6. Multiple sequence alignment of BoVapB1 with previously characterized VapB homologs in other bacteria.** The primary sequence of BoVapB1 (AXW83_01400) was aligned with various VapB homologs in *S. flexneri* (Sf), *S. meliloti* (Sm), *A. bacterium* (Ab), and *B. fragilis* (Bf). The secondary structure of SfVapB was presented above alignment. The blue asterisks represent the conserved aromatic amino acids (W53, F56, and F57) of VapB which are important for interaction with VapC. The numbers correspond to the residues. The GenBank accession numbers for the VapB homologs are as follows: SfVapB; WP_000450531.1, SmVapB; WP_127666133.1, AbVapB; NDA60253.1, and BfVapB; KAA4851477.1.

**Supplemental Figure S7. Viability analyses of cells expressing BoVapC1. (A)** Quantification of the viability of BL21(DE3) expressing BoVapC1. Cells were cultivated as in Fig. 5A and then stained with SYTO9 and PI dye. Fluorescence intensity was measured using a fluorescence spectrophotometer (Hitachi F-7000). The ratio of live to total cells was calculated by dividing the green fluorescence intensity into sum of green and red intensities. Independent experiments were performed in triplicate. Error bars represent SD. **(B)** Growth resumption after BoVapC1 expression. BL21(DE3) cells harboring pBAD33 or pBAD33-BoVapC1 were cultivated to the early exponential phase in M9 minimal media containing Cm at 37℃. The cell cultures were diluted five-fold in fresh M9 media with 0.4% arabinose and incubated at 18℃ for 24 h. At each time point (0, 3, 6, 12, and 24 h), the induced cultures were diluted and spotted onto M9 agar plates with or without arabinose and incubated at 18°C.

**Supplemental Figure S8.** **RNA cleavage activity assay of BoVapC1 at 37°C. (A)** qRT-PCR analyses of mRNAs, rRNAs, and tRNAs. Total RNAs were extracted from BL21(DE3) cells carrying pBAD33 or pBAD33-BoVapC1 after induction for 3 h at 37°C, and these RNAs were utilized for qRT-PCR analyses. Three independent experiments were carried out, and error bars represent SD. NS, non-significant. **(B)** Northern blotting analyses of tRNAs. RNAs extracted from each transformant in (A) before (0 h) and after induction (3 h) at 37°C were analyzed as in Fig. 6B. Full-length tRNAs are indicated with open arrowheads. C indicates pBAD33.

**Supplemental Figure S9. The expression level comparison of *BovapC1* depending on temperature.** BL21(DE3) cells harboring pBAD33-BoVapC1 were cultivated at 37°C to an early exponential phase, followed by induction with 0.4% arabinose. After further incubation for 20 min at 37°C or 60 min at 18°C, cells were harvested by centrifugation. Total RNAs extraction and qRT-PCR analyses were carried out as in Fig. 6. The *hisG* gene served as an endogenous reference. Three independent experiments were carried out, and error bars represent SD. Statistical significance was derived from the two-tailed *t*-test. ∗*p* < 0.05; ∗∗*p* < 0.01. Sistrom WR. 1962. The kinetics of the synthesis of photopigments in Rhodopseudomonas spheroides. *J Gen Microbiol* **28**: 607-616. doi:10.1099/00221287-28-4-607
