# Supplementary material for: Identification and characterization of VapBC toxin–antitoxin system in Bosea sp. PAMC 26642 isolated from Arctic lichens
Source: RNA. 2021 Nov;27(11):1374–89. doi: 10.1261/rna.078786.121 (PMC8522696; doi:10.1261/rna.078786.121)
Supplement: Supplemental Material [file supp_078786.121_Supplemental_Table_S1.docx]

**Supplemental Table S1. Oligonucleotides used in this study.**

| Oligonucleotides | Sequences (5’→3’) | Sources |
| --- | --- | --- |
| Construction of plasmids | | |
| BoVapC1-D7A-5 | TGGCCTATATGCTCGCCACGAATATTTGCATC | This study |
| BoVapC1-D7A-3 | GATGCAAATATTCGTGGCGAGCATATAGGCCA | This study |
| BoVapC1-E42A-5 | GCGTAACTCTGGGAGCGCTGAGCTATGGAGC | This study |
| BoVapC1-E42A-3 | GCTCCATAGCTCAGCGCTCCCAGAGTTACGC | This study |
| BoVapC1-D98A-5 | CTTGCGGGCCGCTCGCCACCCAGATCGGCGC | This study |
| BoVapC1-D98A-3 | GCGCCGATCTGGGTGGCGAGCGGCCCGCAAG | This study |
| BoVapC1-E119A-5 | CCAATAATCGCCGCGCATTTGACCGCATGCC | This study |
| BoVapC1-E119A-3 | GGCATGCGGTCAAATGCGCGGCGATTATTGG | This study |
| BoVapB1-W53R-5 | CCGAAAGGCAAGGGACGGGACGACTTCTTCG | This study |
| BoVapB1-W53R-3 | CGAAGAAGTCGTCCCGTCCCTTGCCTTTCGG | This study |
| BoVapB1-F56SF57S-5 | AGGGATGGGACGACTCCTCCGACAATGGCCCCTT | This study |
| BoVapB1-F56SF57S-3 | AAGGGGCCATTGTCGGAGGAGTCGTCCCATCCCT | This study |
| BoVapB1-W53RF56SF57S-5 | GCCGAAAGGCAAGGGACGGGACGACTCCTCCG | This study |
| BoVapB1-W53RF56SF57S-3 | CGGAGGAGTCGTCCCGTCCCTTGCCTTTCGGC | This study |
| metZWV-U | GAGCAGCCTCTCCCTGAC | This study |
| metZWV-D | GCTGGCGAGAAGGGGATG | This study |
| qRT-PCR | | |
| RT-BoVapC1-5 | TATGGAGCTGAGAAGTCTGAT | This study |
| RT-BoVapC1-3 | GGCGATTATTGGTGACGAC | This study |
| rrlA-F | ATATTCCTGTACTTGGTGTT | (Choi et al. 2020b) |
| rrlA-R | CTTGGTATTCTCTACCTGAC | (Choi et al. 2020b) |
| rrsA-F | GACTTGGAGGTTGTGCCCTT | (Choi et al. 2020b) |
| rrsA-R | GATAAGGGTTGCGCTCGTTG | (Choi et al. 2020b) |
| hisR-F | ATAGCTCAGTTGGTAGAGCCC | This study |
| hisR-R | TGGGGTGGCTAATGGGATTC | This study |
| metU-F | GGTGGCTACGACGGGATTC | This study |
| metU-R | ACGTAGCTCAGTTGGTTAGAGC | This study |
| metV-F | GGGTGGAGCAGCCTGGTA | This study |
| metV-R | CGGGGGCCGGATTTGAA | This study |
| polA-F | AGCTCACCCTTCGTCTGGCT | (Kim et al. 2016) |
| polA-R | AAACGCCTGGCGGATACGAC | (Kim et al. 2016) |
| glyS1-F | CTCTGGCGAAACTGCCGATC | (Kim et al. 2016) |
| glyS1-R | GGCACCGCGAGGAATTTCTC | (Kim et al. 2016) |
| glyS2-F | CACTATGCGCGTCACGATGG | (Kim et al. 2016) |
| glyS2-R | GCCTGGATGGTGTCAACGGT | (Kim et al. 2016) |
| gmd-F | TTGAAGAGAAGGGCATTGTG | (Choi et al. 2020a) |
| gmd-R | GAGATTTCAGCAGAGAGTGT | (Choi et al. 2020a) |
| hisG-F | GTAATGGACGGCGTGGTAG | This study |
| hisG-R | TCTGGTCGAGATAACGCTTG | This study |
| holA-F | ACGACCTGCTGTTGATCGTC | This study |
| holA-R | CCAGACGCAGTTGCTGAAGA | This study |
| recA-F | TCAACTTCTACGGCGAACTG | This study |
| recA-R | CGCCTTCGCTATCATCTACA | This study |
| Northern blotting | | |
| metV-probe | TGGTTGCGGGGGCCGGATTTGA | This study |
| metU-probe | TGGTGGCTACGACGGGATTCGAAC | This study |
| hisR-probe | TGGGGTGGCTAATGGGATTCGAACC | This study |

Choi E, Jeon H, Oh C, Hwang J. 2020a. Elucidation of a Novel Role of YebC in Surface Polysaccharides Regulation of *Escherichia coli bipA*-deletion. *Front Microbiol* **11**. doi:10.3389/fmicb.2020.597515

Choi E, Jeon H, Oh JI, Hwang J. 2020b. Overexpressed L20 Rescues 50S Ribosomal Subunit Assembly Defects of *bipA*-deletion in *Escherichia coli*. *Front Microbiol* **10**. doi:10.3389/fmicb.2019.02982

Kim Y, Choi E, Hwang J. 2016. Functional Studies of Five Toxin-Antitoxin Modules in *Mycobacterium tuberculosis* H37Rv. *Front Microbiol* **7**. doi:10.3389/fmicb.2010.02071
