# Supplementary material for: Identification and characterization of VapBC toxin–antitoxin system in Bosea sp. PAMC 26642 isolated from Arctic lichens
Source: RNA. 2021 Nov;27(11):1374–89. doi: 10.1261/rna.078786.121 (PMC8522696; doi:10.1261/rna.078786.121)
Supplement: Supplemental Material [file supp_078786.121_Supplemental_Table_S2.docx]

**Supplemental Table S2. Bacterial strains and plasmids used in this study.**

| **Strains or plasmids** | **Genotypes** | **Sources** |
| --- | --- | --- |
| *E. coli* DH5α | *fhuA2*, Δ(*lacZYA*-*argF*)U169, *phoA*, *glnV44*, *Φ80'*, *lacZ*Δ*M15*, *gyrA96*, *recA1*, *relA1*, *endA1*, *thi-1*, *hsdR17* | (Jessee 1986) |
| *E. coli* BL21(DE3) | F^-^, *lon-11*, Δ(*ompT-nfrA*)*885,* Δ(*galM-ybJ*)*884*, *λDE3* [*lacI, lacUV5-T7 gene 1*, *ind1, sam7, nin5*], Δ*46*, [*mal^+^*]*_k-12_*(λ^S^), *hsdS10* | (Weiner et al. 1994) |
| *E. coli* S17-1 | *thi* *pro* *recA* *hsdR* [RP4-2Tc::Mu-Km::Tn7] | (Simon et al. 1983) |
| *R. sphaeroides* 2.4.1 | Wild type | (van Niel 1944) |
| pUCIDT-BoVapB1 | *AXW83_01400*^+^ (antitoxin), pUCIDT | This study |
| pUCIDT-BoVapC1 | *AXW83_01405*^+^ (toxin), pUCIDT | This study |
| pUCIDT-BoVapC2 | *AXW83_06920*^+^ (toxin), pUCIDT | This study |
| pUCIDT-BoVapB3 | *AXW83_RS08500*^+^ (toxin), pUCIDT | This study |
| pUCIDT-BoVapC3 | *AXW83_RS26480*^+^ (antitoxin), pUCIDT | This study |
| pUCIDT-BoHicA | *AXW83_10240*^+^ (toxin), pUCIDT | This study |
| pUCIDT-BoHicB | *AXW83_10245*^+^ (antitoxin), pUCIDT | This study |
| pUCIDT-BoHigB1 | *AXW83_11315*^+^ (toxin), pUCIDT | This study |
| pUCIDT-BoVapB4 | *AXW83_11460*^+^ (antitoxin), pUCIDT | This study |
| pUCIDT-BoVapC4 | *AXW83_11465*^+^ (toxin), pUCIDT | This study |
| pUCIDT-BoHigB2 | *AXW83_11955*^+^ (toxin), pUCIDT | This study |
| pUCIDT-BoHigA2 | *AXW83_11960*^+^ (antitoxin), pUCIDT | This study |
| pUCIDT-BoVapB5 | *AXW83_12680*^+^ (antitoxin), pUCIDT | This study |
| pUCIDT-BoVapC5 | *AXW83_12685*^+^ (toxin), pUCIDT | This study |
| pUCIDT-BoFicT | *AXW83_13165*^+^ (toxin), pUCIDT | This study |
| pUCIDT-BoHigB3 | *AXW83_17295*^+^ (toxin), pUCIDT | This study |
| pUCIDT-BoHigA3 | *AXW83_17300*^+^ (antitoxin), pUCIDT | This study |
| pUCIDT-BoHigA4 | *AXW83_18820*^+^ (antitoxin), pUCIDT | This study |
| pUCIDT-BoHigB4 | *AXW83_18825*^+^ (toxin), pUCIDT | This study |
| pUCIDT-BoRelB | *AXW83_26135*^+^ (toxin), pUCIDT | This study |
| pUCIDT-BoRelE | *AXW83_26140*^+^ (antitoxin), pUCIDT | This study |
| pUCIDT-BoVapB1_W53R_ | *AXW83_01400* (W53R), pUCIDT | This study |
| pUCIDT-BoVapB1_F56SF57S_ | *AXW83_01400* (F56SF57S), pUCIDT | This study |
| pUCIDT-BoVapB1_W53RF56SF57S_ | *AXW83_01400* (W53RF56SF57S), pUCIDT | This study |
| pBAD33 | *araBAD* promoter, pACYC184 *ori*, Cm^R^ | (Guzman et al. 1995) |
| pBAD33-BoVapC1 | *AXW83_01405*^+^, pBAD33 | This study |
| pBAD33-BoVapC2 | *AXW83_06920*^+^, pBAD33 | This study |
| pBAD33-BoVapC3 | *AXW83_RS08500*^+^, pBAD33 | This study |
| pBAD33-BoHicA | *AXW83_10240*^+^, pBAD33 | This study |
| pBAD33-BoHigB1 | *AXW83_11315*^+^, pBAD33 | This study |
| pBAD33-BoVapC4 | *AXW83_11465*^+^, pBAD33 | This study |
| pBAD33-BoHigB2 | *AXW83_11955*^+^, pBAD33 | This study |
| pBAD33-BoVapC5 | *AXW83_12685*^+^, pBAD33 | This study |
| pBAD33-BoFicT | *AXW83_13165*^+^, pBAD33 | This study |
| pBAD33-BoHigB3 | *AXW83_17295*^+^, pBAD33 | This study |
| pBAD33-BoHigB4 | *AXW83_18825*^+^, pBAD33 | This study |
| pBAD33-BoRelB | *AXW83_26135*^+^, pBAD33 | This study |
| pBAD33-BoVapC1_D7A_ | *AXW83_01405* (D7A), pBAD33 | This study |
| pBAD33-BoVapC1_E42A_ | *AXW83_01405* (E42A), pBAD33 | This study |
| pBAD33-BoVapC1_D98A_ | *AXW83_01405* (D98A), pBAD33 | This study |
| pBAD33-BoVapC1_E119A_ | *AXW83_01405* (E119A), pBAD33 | This study |
| pBAD33-His_6_-BoVapB1 | *His_6_-AXW83_01400*^+^, pBAD33 | This study |
| pRK415 | Tc^R^, *oriV* RK2, *oriT* RK2 | (Keen et al. 1988) |
| pRK-BAD | pBAD33 cloned into pRK415 | This study |
| pRK-BAD-BoVapC1 | *AXW83_01405*^+^, pRK-BAD | This study |
| pRK-BAD-BoVapC2 | *AXW83_06920*^+^, pRK-BAD | This study |
| pRK-BAD-BoVapC3 | *AXW83_RS08500*^+^, pRK-BAD | This study |
| pRK-BAD-BoHicA | *AXW83_10240*^+^, pRK-BAD | This study |
| pRK-BAD-BoHigB1 | *AXW83_11315*^+^, pRK-BAD | This study |
| pRK-BAD-BoVapC4 | *AXW83_11465*^+^, pRK-BAD | This study |
| pRK-BAD-BoHigB2 | *AXW83_11955*^+^, pRK-BAD | This study |
| pRK-BAD-BoVapC5 | *AXW83_12685*^+^, pRK-BAD | This study |
| pRK-BAD-BoFicT | *AXW83_13165*^+^, pRK-BAD | This study |
| pRK-BAD-BoHigB3 | *AXW83_17295*^+^, pRK-BAD | This study |
| pRK-BAD-BoHigB4 | *AXW83_18825*^+^, pRK-BAD | This study |
| pRK-BAD-BoRelB | *AXW83_26135*^+^, pRK-BAD | This study |
| pET21c | T7 promoter, pBR322 *ori*, Amp^R^ | Novagen |
| pET21c-BoVapB1 | *AXW83_01400*^+^, pET21c | This study |
| pET21c-BoVapB3 | *AXW83_RS26480*^+^, pET21c | This study |
| pET21c-BoHicB | *AXW83_10245*^+^, pET21c | This study |
| pET21c-BoVapB4 | *AXW83_11460*^+^, pET21c | This study |
| pET21c-BoHigA2 | *AXW83_11960*^+^, pET21c | This study |
| pET21c-BoVapB5 | *AXW83_12680*^+^, pET21c | This study |
| pET21c-BoHigA3 | *AXW83_17300*^+^, pET21c | This study |
| pET21c-BoHigA4 | *AXW83_18820*^+^, pET21c | This study |
| pET21c-BoRelE | *AXW83_26140*^+^, pET21c | This study |
| pET21c-BoVapB1_W53R_ | *AXW83_01400* (W53R), pET21c | This study |
| pET21c-BoVapB1_F56SF57S_ | *AXW83_01400* (F56S, F57S), pET21c | This study |
| pET21c-BoVapB1_W53RF56SF57S_ | *AXW83_01400* (W53R, F56S, F57S), pET21c | This study |
| pET21c-BoVapC1 | *AXW83_01405*^+^, pET21c | This study |
| pET21c-BoVapC1_D7A_ | *AXW83_01405* (D7A), pET21c | This study |
| pET21c-BoVapC1_E42A_ | *AXW83_01405* (E42A), pET21c | This study |
| pET21c-BoVapC1_D98A_ | *AXW83_01405* (D98A), pET21c | This study |
| pET21c-BoVapC1_E119A_ | *AXW83_01405* (E119A), pET21c | This study |
| pBR322 | Amp^R^, Tc^R^ | (Bolivar et al. 1977) |
| pBR322-metZWV | *metZWV*, pBR322 | This study |

Amp^R^; Ampicillin resistance, Cm^R^; Chloramphenicol resistance, Tc^R^; Tetracycline resistance

Bolivar F, Rodriguez RL, Greene PJ, Betlach MC, Heyneker HL, Boyer HW, Crosa JH, Falkow S. 1977. Construction and characterization of new cloning vehicles. II. A multipurpose cloning system. *Gene* **2**: 95-113.

Guzman LM, Belin D, Carson MJ, Beckwith J. 1995. Tight regulation, modulation, and high-level expression by vectors containing the arabinose PBAD promoter. *J Bacteriol* **177**: 4121-4130. doi:10.1128/jb.177.14.4121-4130.1995

Jessee J. 1986. New subcloning efficiency competent cells:> 1× 10^6^ transformants/μg. *Focus* **8**: 1146-1157.

Keen NT, Tamaki S, Kobayashi D, Trollinger D. 1988. Improved broad-host-range plasmids for DNA cloning in Gram-negative bacteria. *Gene* **70**: 191-197. doi:10.1016/0378-1119(88)90117-5

Simon R, Priefer U, Puhler A. 1983. A Broad Host Range Mobilization System for *In vivo* Genetic-Engineering: Transposon Mutagenesis in Gram Negative Bacteria. *Bio-Technol* **1**: 784-791. doi:10.1038/nbt1183-784

van Niel CB. 1944. The Culture, General Physiology, Morphology, and Classification of the Non-Sulfur Purple and Brown Bacteria. *Bacteriol Rev* **8**: 1-118. doi:10.1128/br.8.1.1-118.1944

Weiner M, Anderson C, Jerpseth B, Wells S, Johnson-Browne B, Vaillancourt P. 1994. Studier pET system vectors and hosts. *Strateg Mol Biol* **7**: 41-43.
