# Supplementary material for: Identification and characterization of VapBC toxin–antitoxin system in Bosea sp. PAMC 26642 isolated from Arctic lichens
Source: RNA. 2021 Nov;27(11):1374–89. doi: 10.1261/rna.078786.121 (PMC8522696; doi:10.1261/rna.078786.121)
Supplement: Supplemental Material [file supp_078786.121_Supplemental_Table_S3.docx]

**Supplemental Table S3. The predicted type II TA systems in *Bosea* species.**

| **Species** | **Habitat** | **The number of TA systems** |
| --- | --- | --- |
| *Bosea lathyri* | Root nodules of *Lathyrus latifolius* | 0 |
| *Bosea lupini* | Agriculture soil | 0 |
| *Bosea thiooxidans* | Agriculture soil | 0 |
| *Bosea robiniae* | Legumes | 0 |
| *Bosea caraganae* | Root nodules of the relict species *Caragana jubata* | 2 |
| *Bosea vestrisii* | Hospital water supplies | 2 |
| *Bosea psychrotolerans* | Lake Michigan water | 5 |
| *Bosea* sp. PAMC 26642 | An Arctic lichen | 12 |
